# Biological Activity of Novel Organotin Compounds with a Schiff Base Containing an Antioxidant Fragment

**DOI:** 10.3390/ijms24032024

**Published:** 2023-01-19

**Authors:** Taisiya A. Antonenko, Yulia A. Gracheva, Dmitry B. Shpakovsky, Mstislav A. Vorobyev, Dmitrii M. Mazur, Victor A. Tafeenko, Yury F. Oprunenko, Elena F. Shevtsova, Pavel N. Shevtsov, Alexey A. Nazarov, Elena R. Milaeva

**Affiliations:** 1 Department of Chemistry, M. V. Lomonosov Moscow State University, Leninskie Gory 1/3, 119991 Moscow, Russia; 2 Institute of Geography of the Russian Academy of Sciences, Department of Glaciology, 117312 Moscow, Russia; 3 Institute of Physiologically Active Compounds of Russian Academy of Sciences, 142432 Chernogolovka, Russia

**Keywords:** organotin complexes, Schiff base, antioxidant activity, antiproliferative activity, apoptosis, cell cycle

## Abstract

A series of novel organotin(IV) complexes on the base of 2-(N-3′,5′-di-*tert*-butyl-4′-hydroxyphenyl)-iminomethylphenol (**L**) of formulae Me_2_SnBr_2_(L)_2_ (**1**), Bu_2_SnCl_2_(L)_2_(**2**), Ph_2_SnCl_2_(L) (**3**), Ph_2_SnCl_2_(L)_2_ (**4**) Ph_3_SnBr(L)_2_ (**5**) were synthesized and characterized by ^1^H, ^13^C, ^119^Sn NMR, IR, ESI-MS and elemental analysis. The crystal structures of initial **L** and complex **2** were determined by XRD method. It was found that **L** crystallizes in the orthorhombic syngony. The distorted octahedron geometry around Sn center is observed in the structure of complex **2**. Intra- and inter-molecular hydrogen bonds were found in both structures. The antioxidant activity of new complexes as reducing agents, radical scavengers and lipoxygenase inhibitors was estimated spectrophotometrically in CUPRAC and DPPH tests (compounds **1** and **5** were found to be the most active in both methods), and in the process of enzymatic oxidation in vitro of linoleic acid under the action of lipoxygenase LOX 1-B (EC_50_ > 33.3 μM for complex **2**). Furthermore, compounds **1–5** have been investigated for their antiproliferative activity in vitro towards HCT-116, MCF-7 and A-549 and non-malignant WI-38 human cell lines. Complexes **2** and **5** demonstrated the highest activity. The plausible mechanisms of the antiproliferative activity of compounds, including the influence on the polymerization of Tb+MAP, are discussed. Some of the synthesized compounds have also actively induced apoptosis and blocked proliferation in the cell cycle G2/M phase.

## 1. Introduction

It is known that platinum compounds were the first drugs based on metal compounds that were widely used in cancer treatment therapy. Nevertheless, they are highly toxic and show poor selectivity for different cell lines, and many types of cancer have developed resistance to them. Therefore, the search for new organometallic compounds with anti-tumor activity is an urgent task [1,2,3]. A significant part of this large class of compounds is represented by organotin complexes. It is known that tin complexes with coordination number >4 have a wide spectrum of biological activity, such as fungicidal, antibacterial, nematicidal, insecticidal, herbicidal, anti-inflammatory and anti-tumor activities [4]. It was found that the structure of anti-tumor drugs based on tin compounds is characterized by the presence of a stable ligand-metal bond resistant to hydrolysis. Higher cytotoxicity is manifested when the coordination environment of the tin atom is completely filled (CN = 6) and when the Sn–N and Sn–S bonds are the shortest [5].

Schiff bases common organic ligands. They can be applied as dyes, catalysts, intermediates, polymer stabilizers and pigments. These compounds also demonstrate various types of biological activities, e.g., antimalarial, anti-inflammatory, antiviral and antiproliferative ones [6]. Complexes of Fe(III), Co(II), Zn(II), and Zr(IV) with Schiff base gemifloxacin exhibited promising antifungal activity [7]. Also, there is a study reporting significant biological activity of enrofloxacin Schiff base complexes (H_2_Erx-en) of Fe(III), Y(III), Zr(IV) and La(III) that demonstrated extremely significant antibacterial activity data [8]. The Cu (II) complex with Gat-o-phdn Schiff base (4E,4′E)-4,4′-(1,2-phenylenebis (azaneylylidene))bis(1-cyclopropyl-6-fluoro-8-methoxy-7-(3-methylpiperazin-1-yl)-1,4-dihydroquinoline-3-carboxylic acid demonstrated very high antimicrobial activity against *Staphylococcus aureus* [9].

One of the proposed mechanisms of organotins’ action may be due to Sn atom’s ability to bind to sulfhydryl groups, followed by DNA destructionthat ultimately leads to cell death [10]. The toxicity of organotins can also induce oxidative stress in living organisms. To decrease their unwanted high toxicity, it is proposed to use antioxidants; 2,6-dialkylphenols are known to be vitamin E mimetics and inhibitors of radical oxidative processes [11,12]. We found previously that organotin complexes containing Schiff base with 2,6-dialkylphenol fragment demonstrate both good antioxidant and cytotoxic activities [13].

**Scheme 1:**
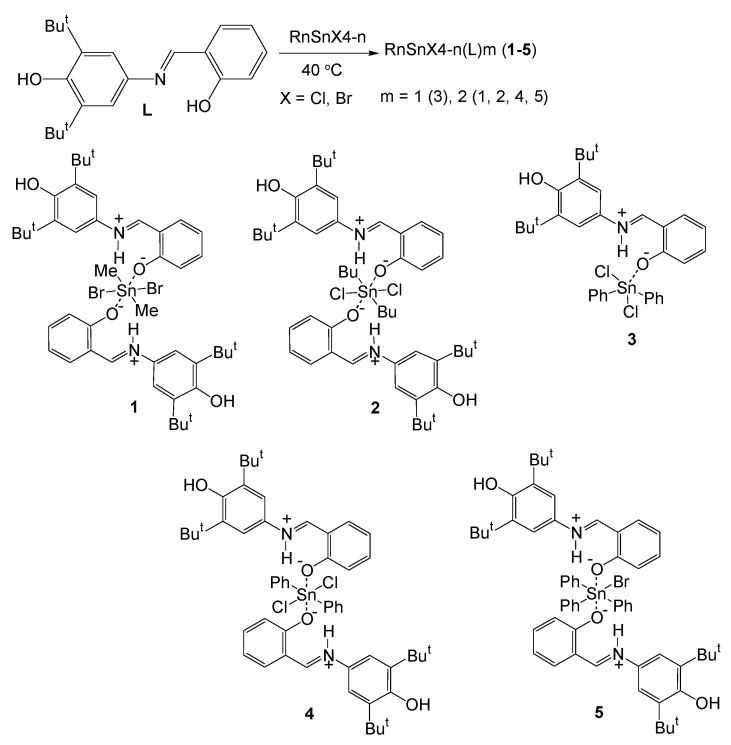
Synthesis of organotin complexes **1**–**5** based on **L**.

The purpose of this work was the synthesis of novel organotin complexes **1**–**5** with Schiff base **L** containing an antioxidant fragment of 2,6-di-*tert*-butylphenol (Scheme 1) and the study of their biological activity. Herein we report the antioxidant and antiproliferative properties of new complexes **1**–**5**.

## 2. Results and Discussion

### 2.1. Synthesis and Characterization

Novel complexes **1**–**4** were obtained by the interaction of **L** with organotin chlorides Bu_2_SnCl_2_, Ph_2_SnCl_2_ and bromide Me_2_SnBr_2_ (Scheme 1) in methylene chloride with stirring and low heating (40 °C). The new complex **5** (Scheme 1) was synthesized by the interaction of **L** with Ph_3_SnBr in a mixture of chloroform and ethanol. Compounds **1**–**5** were found to be stable in air and were explored by elemental analysis, IR, ^1^H, ^13^C and ^119^Sn NMR spectroscopy.

The IR, ^1^H and ^13^C spectroscopic data (Figures S1–S10) are comparable with those for structurally similar complexes [13].The IR spectra of compounds **1**–**5** demonstrate absorption bands in the region of 3492–3643 cm^−1^, appropriating to the stretching vibrations of the O-H bond of the sterically hindered non-associated phenolic group.

The proton signal of the phenol group is absent in the ^1^H NMR spectra of complexes **1**–**5**, whereas for the starting compound **L** the signal is observed at 13.64 ppm due to solvent exchange. The spin-spin interaction of H-Sn (Ph_2_Sn fragment,^2^J_Sn-H_ = 80 Hz) is also found in the ^1^H NMR spectrum of complex **4**.

The chemical shifts in ^119^Sn spectra varied in the wide interval (from −326 to +112 ppm) depending on the environment of Sn atom (Figures S11–S15).

**Figure 1:**
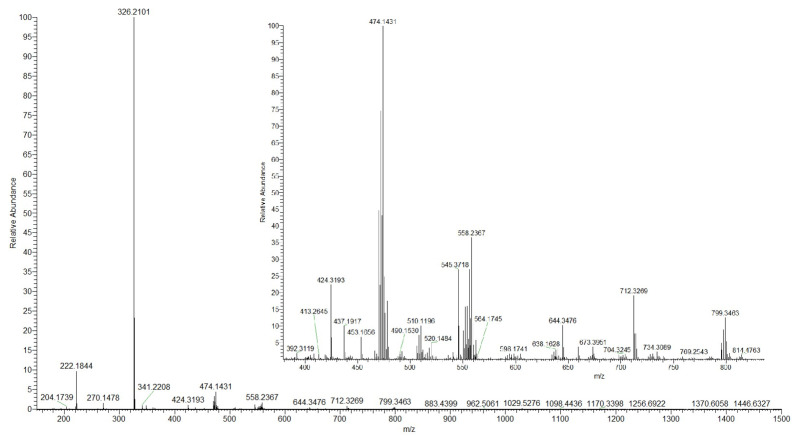
ESI mass spectrum of compound **1**.

Compounds **1**–**5** were also characterized by HRMS. It turns out that during electrospray ionization, the complex structure is broken, and the charge stays on the ligand part only. There is however indirect evidence, which is described below, in favor of the proposed structure coming from HRMS data. It appears that some intermolecular reactions or redox reactions besides just ionization processes may take place during ESI. The ligand environment, hence, could be significantly changed through the loss of some ligands or by substitution reactions. Compound **1** forms ions *m*/*z* 326.2101 C_21_H_28_O_2_N, *m*/*z* 474.1431 C_23_H_32_O_2_NSn (Me_2_SnL), *m*/*z* 799.3463 C_44_H_59_O_4_N_2_Sn (Me_2_SnL_2_) (Figure 1). Base peak *m*/*z* 326.2104 (C_21_H_28_O_2_N) corresponds to the protonated molecule of ligand.

Similar ions are observed in the mass spectrum of compound **2** with *m*/*z* 326.2101 C_21_H_28_O_2_N (L), *m*/*z* 558.2370 C_29_H_44_O_2_NSn (Bu_2_SnL), *m*/*z* 594.2129 C_29_H_45_O_2_NClSn (Bu_2_SnClL), *m*/*z* 883.4399 C_50_H_71_O_4_N_2_Sn (Bu_2_SnL_2_) (Figure S16).

In the case of compound **3** two clusters of ions are observed at *m*/*z* 594–602 and *m*/*z* 672–680, which the isotopic distribution and accurate masses of which nicely fit the formulae.C_33_H_36_O_2_NSn (Ph_2_SnL) and C_39_H_42_O_2_NSn (Ph_3_SnL) correspondingly, result from loss of chloride ligands. Signals of low abundance within *m*/*z* 612–636 were assigned for C_33_H_37_O_2_NClSn (Ph_2_SnClL) and C_33_H_38_O_3_NSn (Ph_2_SnOHL) (Figure S17).

The base peak in the mass spectrum of compound **4** is similar tothat for **3** since they both have the same ligand (Figure S18). However, together with the ions mentioned above there are signals in the *m*/*z* 800–950 range (e.g., *m*/*z* 923.3742 corresponding to Ph_2_SnL_2_, i.e._,_ C_54_H_63_O_4_N_2_Sn) bringing evidence to the *bis*-ligand nature of the compound **4**. Substitution of Cl-ligands for Br doesn’t significantly change the mass spectrum of compound **5** (Figure S19). The most abundant peaks are the same. The bromine atom is missing in observed signals, but all the rest of the moieties are revealed: *m*/*z* 598.1732 C_33_H_36_O_2_NSn (Ph_2_SnL), *m*/*z* 676.2203 C_39_H_42_O_2_NSn (Ph_3_SnL), *m*/*z* 854.4114 C_48_H_66_O_4_N_2_Sn, *m*/*z* 923.3744 C_54_H_63_O_4_N_2_Sn (Ph_2_SnL_2_). Though the studied complexes did not form any molecular species ([M+H]^+^, [M+Na]^+^, etc.) under ESI conditions, the ions described above still are useful and reveal the proposed molecular structure.

### 2.2. Crystal Structures

Recrystallization of compound **L** from a mixture of petroleum ether and CH_2_Cl_2_ gave orange crystals, which were used for crystallographic analysis (Table 1).

It has been established that the ligand crystallizes in the orthorhombic syngony. The N1-C7 bond distance is 1.272(3) Å. X-ray diffraction analysis (XRD) showed the presence of an intramolecular hydrogen bond O2-H32^…^N1 (H32^…^N1 = 1.47 Å, O2-H32^…^N1= 2.54 Å, angle O2-H32-N1 = 150.95°), as well as an intermolecular hydrogen bond O1-H31^…^O2 (H31^…^O2 = 2.13 Å, O1-H31^…^O2 = 2.80 Å, angle O1-H31^-^O2 = 137.23°).

**Figure 2:**
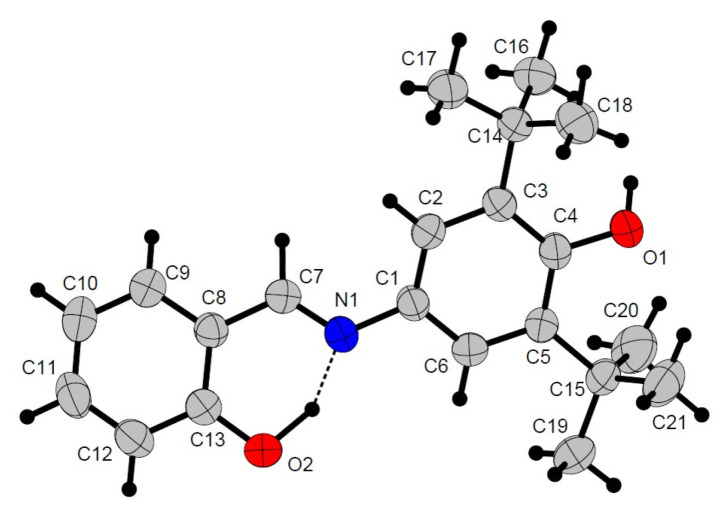
Molecular structure of starting compound **L**.

The angle between the ring plane of the 2,6-di-*tert*-butylphenol fragment (C1C2C3C4C5C6) and the ring plane of the unhindered phenol group (C8C9C10C11C12C13) is 22.26°. The molecular structure of the compound is shown in Figure 2. Table 2 demonstrates the individual bond distances and angles of **L**.

**Figure 3:**
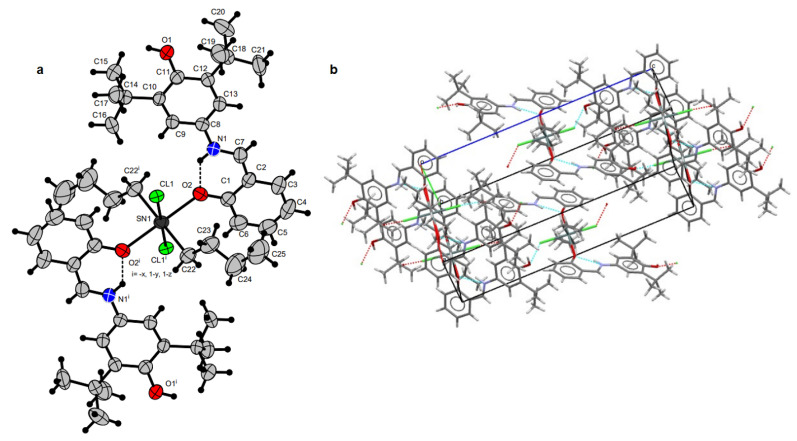
Molecular structure of **2** (**a**); hydrogen bonds in crystal packing **2** (**b**).

As a result of the recrystallization of compound **2** from hexane, orange single crystals, suitable for XRD, were obtained. The coordination number around the tin atom in crystals (L)_2_Bu_2_SnCl_2_ (**2**) is 6; the coordination polyhedron is a distorted octahedron. The butyl groups, chlorine atoms, and oxygen atoms of the unhindered phenolic group are in a trans-position with respect to each other in the inner coordination sphere of the tin atom (Figure 3a).

Due to proton transfer from the oxygen atom O2 to the nitrogen atom N1, intramolecular hydrogen bonds N1–H21⋯O2 are formed in the structure (H21⋯O2 = 1.80 Å, N1–H21⋯O2 = 2.58 Å, angle N1–H21-O2 = 137.85°) (Figure 3b). It should be noted that, in comparison with the initial ligand, in the structure of the obtained complex, the angle between the ring plane of the 2,6-di-*tert*-butylphenol fragment and the ring plane of the unhindered phenol group in the structure of **2** decreases significantly and amounts to 4.50°, i.e., these fragments lie almost in the same plane. The individual bond distances and angles of **2** are listed in Table 2.

### 2.3. CUPRAC Assay (Cu^2+^ Reducing)

One of the methods for studying the antioxidant activity of compounds is to research their ability to one-electron reduction using the spectrophotometric CUPRAC test. Neocuproine (2,9-dimethyl-1,10-phenanthroline) forms a complex with Cu^+^ in the presence of antioxidants with an absorption maximum at 450 nm [14]. The experiment was carried out to increase the optical density of a solution of the complex in ethanol. Trolox (6-hydroxy-2,5,7,8-tetramethylchroman-2-carboxylic acid) was used as a standard. The results are presented as TEAC values (Trolox equivalents antioxidant capacity, Table 3). TEAC for Trolox is 1.00 ± 0.03. It was shown that among the most active complexes are compounds **1**, **2**, **4** and **5**.

### 2.4. DPPH Radical Scavenging Activity

A method for studying the antioxidant (radical scavenging) activity of compounds is their ability to reduce the stable 2,2-diphenyl-1-picrylhydrazyl (DPPH) radical by hydrogen atom transfer [15].

The activity of tin compounds **1**–**5** in the DPPH test, as well as the initial ligand **L**, was studied spectrophotometrically by measuring the decrease in the optical density of DPPH at a wavelength of 517 nm for 1 h. The value of the EC_50_ parameter (the effective concentration of the compound required to reduce the concentration of the DPPH radical by 50%) was determined graphically according to the dependence of the content of the remaining DPPH (in %) on the primary concentration of compounds (0.01–0.1 mM). EC_50_ values are presented in Table 4.

Since compounds **1**–**5** showed rather high activity, their reaction kinetics were studied, which corresponds to the second order equation. The rate constants *k* (for each concentration) are obtained from the plot of 1/[DPPH] vs. time (Table 4). It is known that *k* for **L** at a concentration of 0.06 mM is 10.9 L mol^−1^ s^−1^ [13]. At concentrations higher than 0.04 mM the rate of the DPPH reduction was too high to be evaluated.

### 2.5. Lipoxygenase Inhibition Assessment

Lipoxygenase LOX 1-B is a plant enzyme that belongs to the class of iron-containing oxygenases and catalyzes the stereospecific oxidation of polyunsaturated fatty acids in the cell, including linoleic acid. It is known that lipoxygenase is able to participate in the destruction of biomembranes [16]. Therefore, the ability of a compound to inhibit this enzyme may indicate its potential antioxidant and anti-inflammatory properties. The antioxidant properties of the obtained compounds were also evaluated in the process of enzymatic oxidation of linoleic acid under the action of lipoxygenase LOX 1-B in vitro. The experiment was carried out by spectrophotometry measuring the content of the oxidation product of linoleic acid—the corresponding hydroperoxides at λ_max_ 234 nm. It was found that the majority of the compounds promote the oxidation of linoleic acid, while complex **2** is only a moderate inhibitor of lipoxygenase (EC_50_ > 33.3 μM). The results are listed in Table 5.

### 2.6. Antiproliferative Activity

The antiproliferative activity of compounds **1**–**5** was also studied. The compounds were investigated against HCT-116 (human colon cancer), MCF-7 (human breast cancer), A-549 (adenocarcinoma human alveolar basal epithelial) and WI-38 (diploid human cell line composed of fibroblasts) cells using the MTT test [17]. The IC_50_ values were determined in comparison with cisplatin (Table 6).

It is known that, depending on the organic substituent nature, the cytotoxicity of organotin compounds R_n_SnX_4-n_L decreases in accordance with *n*-Bu > Ph, Et > Me [18,19].

**Figure 4:**
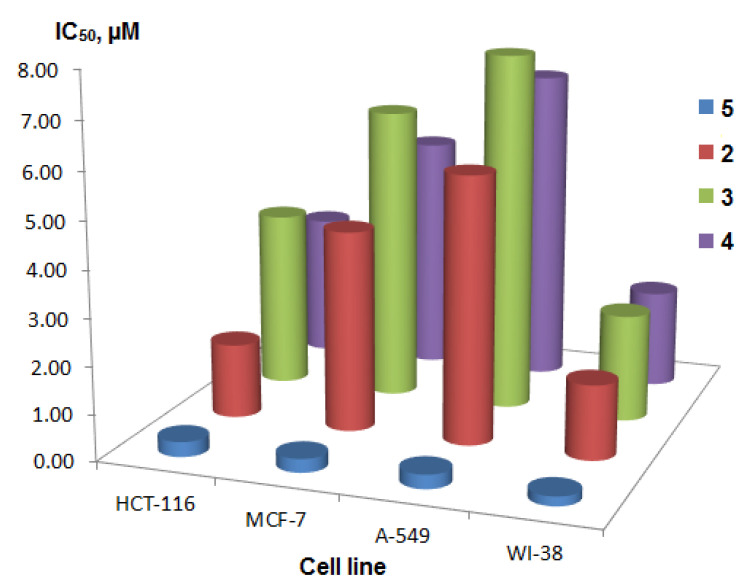
Survival of cell lines HCT-116, MCF-7, A-549 and WI-38 in the presence of compounds **2**–**5**.

Among the obtained tin complexes, the triphenyltin complex **5** exhibits nanomolar activity (Table 6) that is consistent with the results of our previous works concerning triphenyltin complexes based on 2,6-di-*tert*-butyl-4-mercaptophenol which exhibited the high activity related with the high lipophilicity of Ph_3_Sn fragment. In addition, complexes based on dibutyltin and diphenyltin **2**–**4** also demonstrate high antiproliferative activity (Figure 4). The activity of obtained complexes against nonmalignant cells WI-38 is comparable to that of HCT-116 cancer cells.

### 2.7. Cell Death and Cell Cycle Analysis

**Figure 5:**
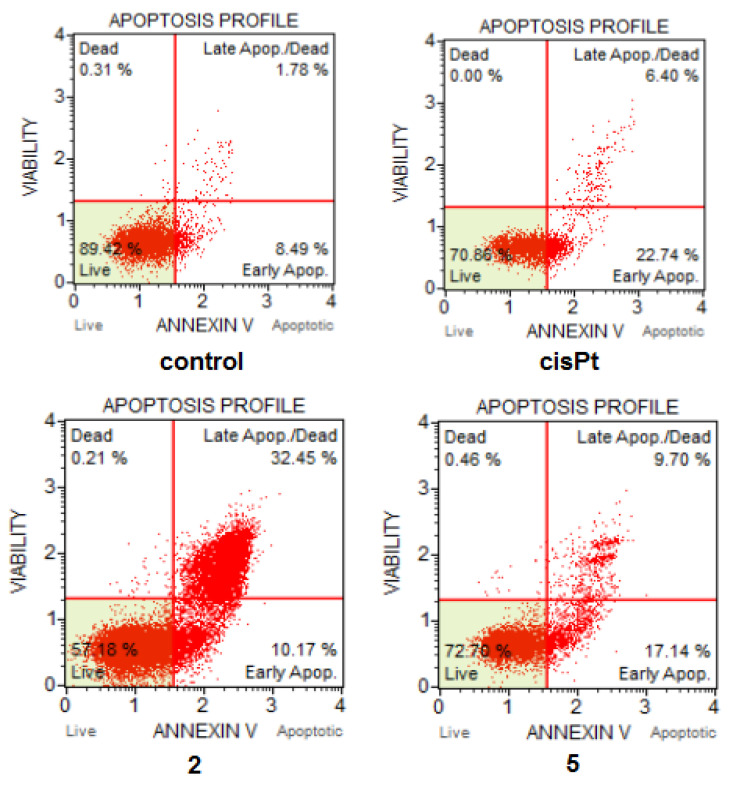
HCT-116 apoptotic profile after treatment with cisplatin and compounds **2**, **5** after 24 h. Concentration of compounds 2·IC_50_ (µM).

Apoptosis, known as programmed cell death, is a carefully controlled, energy-dependent process. Cells initiate intracellular processes by responding to specific induction signals. This leads to characteristic physiological changes, which include the externalization of phosphatidylserine (PS) to the cell surface. PS is a membrane component that is usually localized on the inside of the cell membrane. At the beginning of the apoptotic pathway, PS molecules move to the outer surface of the cell membrane, where they can easily be bound by annexin V, a calcium-dependent phospholipid-binding protein with a high affinity for PS. To prove the hypothesis that antiproliferative activity correlates with the induction of apoptosis by organotin compounds, we studied the apoptotic profile of selected cancer cells. The mechanism of cell death was monitored as Annexin V/7-AAD reactivity for compounds **2** and **5** in HCT-116 cells using the Muse^®^AnnexinV& Dead Cell Kit (Luminex Corp., Austin, TX, USA) by flow cytometry [20]. Cells were incubated with compounds and cisplatin for 24 and 48 h at concentrations corresponding to 2·IC_50_ values. Both compounds were shown to induce apoptosis actively. The overall percentage of apoptotic cells is 42.6 and 26.8% for **2** and **5**, respectively. Interestingly, for compound **2** the main fraction of cells was found in late apoptosis (32.4%). The percentage of apoptotic cells did not exceed 10.3% in the control group (Figure 5). Thereby, HCT-116 cells turned out to be more sensitive to the action of compound **2** after 24 h of incubation.

**Figure 6:**
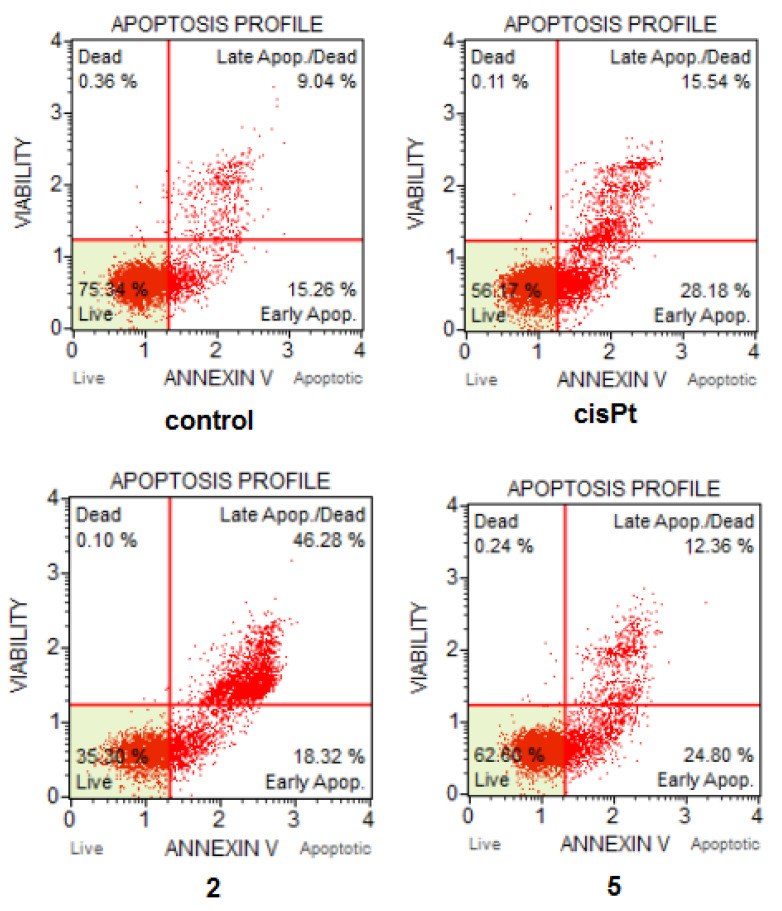
HCT-116 apoptotic profile after treatment with cisplatin and compounds **2**, **5** after 48 h. Concentration of compounds 2·IC_50_ (µM).

An overall increase in apoptotic cells for studied compounds was observed after 48 h, e.g., for **2** their number approaches 65%. The fraction of cells in late apoptosis was 46.3% for **2** (Figure 6).

The cell cyclecan be considered the smallest part of the life cycle. It is the series of steps of growth and development that a cell goes through between its birth and reproduction—dividing to form two new daughter cells. For cell division, several tasks must be taken: the cell must grow (G1 phase), copy its genetic material (S phase), prepare to divide (G2 phase), and divide (by mitosis, or M phase).

The assay uses propidium iodide (PI) based staining of DNA content to discriminate and measure the percentage of cells in each cell cycle phase (G0/G1, S, and G2/M). PI discriminates cells at different stages of the cell cycle, based on differential DNA content in the presence of RNAse to increase the specificity of DNA staining.

**Figure 7:**
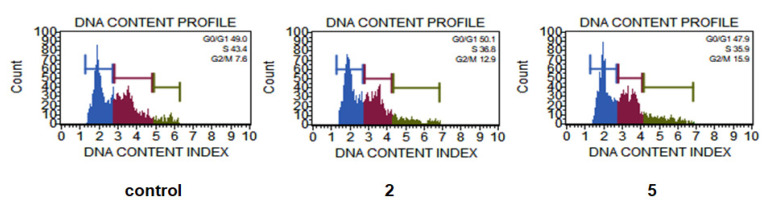
HCT-116 cell cycle analysis after treatment with compounds **2** and **5**. Concentration of compounds IC_50_ (µM).

The effect of compounds **2** and **5** on cell cycle arrest by flow cytometry was carried out. HCT-116 cells were treated for 24 h with compounds at the concentrations of IC_50_. It was found that compounds block proliferation in the G2/M phase of the cell cycle, preparation for mitosis-mitosis (Figure 7).

### 2.8. Tubulin Polymerization

It is known that microtubules and their main component tubulin are the prospectivetargets of antiproliferative chemotherapy drugs. In our previous works [11,21] we demonstrated that cytotoxic organotin complexes containing 2,6-di-*tert*-butylphenol moieties are able to interact with tubulin SH groups. These results suggest that the antiproliferative anti-tumor effects of these compounds may be associated with the disruption of microtubule assembly due to the inhibition of tubulin polymerization in the presence of organotin complexes.

**Figure 8:**
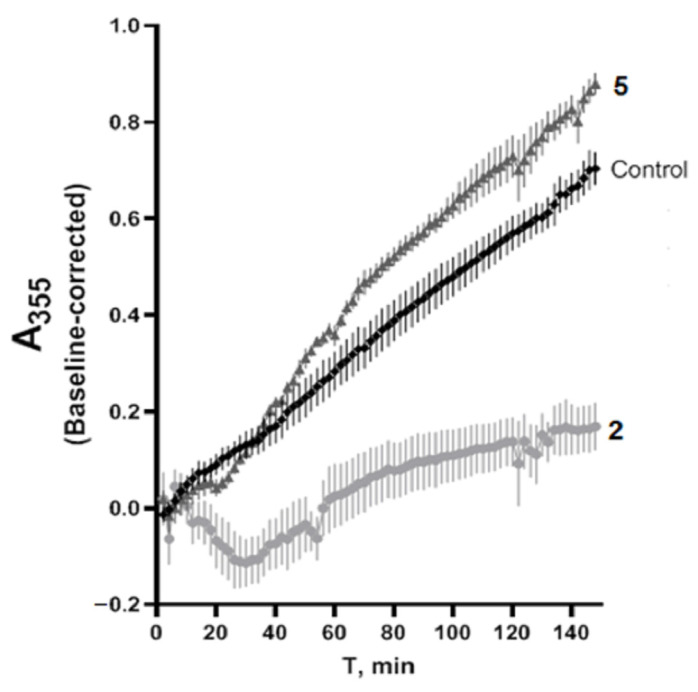
The influence of compounds **2** and **5** (100 µM) on Tb+MAP polymerization. Results presented as mean ± SD obtained using at least from three independent experiments.

In this work, we studied the influence of the most active compounds **2** and **5** on the crude preparation of Tb+MAPpolymerization. It was found that both compounds affect the guanosine triphosphate (GTP) dependent polymerization of Tb+MAP; some stimulation of polymerization is observed for compound **5** and for compound **2** there is a significant blockade of this process that indicates a potential antiproliferative activity (Figure 8). Both stimulation and blockade of the process of tubulin polymerization can be mechanisms of the antiproliferative activity of the compounds, disrupting the dynamic processes of the cell division microtubules spindle [22].

## 3. Materials and Methods

### 3.1. Reagents and Materials

All solvents used were of reagent grade and starting organotin compounds Bu_2_SnCl_2_, Ph_2_SnCl_2_, Me_2_SnBr_2_,Ph_3_SnBr (Sigma-Aldrich, Merck KGaA, Darmstadt, Germany, 98%), Trolox (Acros Organics, Geel, Belgium, 97%) were used as supplied. DPPH, neocuproine (2,9-dimethyl-1,10-phenanthroline, 99%) xanthine, EDTA, nitroblue tetrazolium (NBT), xanthine oxidase (0.04 MU) and bovine serum albumin were bought from Sigma-Aldrich.

Infrared absorption spectra were registered using KBr pellets on IR200 (Thermo Nicolet Corporation, Madison, WI, USA) spectrophotometer with Fourier transform. The NMR spectra were obtained using a Bruker Avance-400 (Brucker, Karlsruhe, Germany) spectrometer at different frequencies: 400.1 MHz (^1^H), 100.6 MHz (^13^C) and 149.15 MHz (^119^Sn) in CDCl_3_and DMSO-d_6_. Orbitrap Elite (Thermo Fisher Scientific, Waltham, MA, USA) mass-spectrometer equipped with an electrospray ionization source (ESI) was used for all high-resolution mass-spectrometry (HRMS) experiments. All compounds dissolved in methanol were directly introduced into the ionization source by means of a syringe pump at 5 μL/min. ESI mass spectra were recorded using positive ionization mode with spray voltage set to 3.4 kV. Xcalibur software (Thermo Xcalibur 3.0.63, Thermo Scientific, Waltham, MA, USA)was used as a system control tool as well as for data collection and data processing. Vaporizer and ion transfer tube temperatures were set to 40 °C and 275 °C correspondingly. All spectra were recorded during 30 s in the *m*/*z* range 150–1500. Elemental analyses were carried out using a Vario Micro Cube analyzer (Elementar, Berlin, Germany). The determination of the antioxidant activity of the compounds was investigated on an Evolution 300 UV-Visible (Thermo Scientific, Waltham, MA, USA) cuvette spectrophotometer and a microplate (96 wells) spectrophotometer Multiskan Go (Thermo Fisher Sci., Waltham, MA, USA). The MTT test was carried out on a Zenyth200rt (Anthos, Biochrom, Cambridge, UK) multiwall-plate reader.

### 3.2. Synthesis

Schiff base 2-(-N-3,5-di-*tert*-butyl-4-hydroxyphenyl)iminomethylphenol (**L**), was obtained according to the well-known method [23]. Synthesis of complexes **1**–**5** is demonstrated in Scheme 1.

#### 3.2.1. Synthesis and Characterization of Me_2_SnBr_2_(L)_2_ (**1**)

A solution of 25 mg (0.07 mmol) Me_2_SnBr_2_ in 2 mL of CH_2_Cl_2_was added to a solution of 50.9 mg (0.148 mmol) of **L** in 3 mL of CH_2_Cl_2_. Then the mixture was stirred at 40 °C for 4 h. The precipitate formed was separated and rinsed with petroleum ether followed by air drying for 24 h. Yield 75 mg (73%);orange-yellow powder; m.p. 135–137 °C. Anal. Calcd for C_44_H_60_N_2_O_4_Br_2_Sn (959.47) (%): C, 55.08; H, 6.30; N, 2.92. Found (%): C, 55.32; H, 6.43; N, 2.78. IR(KBr, ν, cm^−1^): ν(OH) 3627 (m); ν(C-H) 2873–2962 (s); ν(C=N) 1632 (m); 1608; 1486; 1432; 1240; 1157; 766. ^1^HNMR (CDCl_3_, 400.1 MHz, *δ*, ppm): 1.38 (s, 6H, Sn(CH_3_)_2_, ^2^*J_Sn-H_* = 64 Hz); 1.49 (s, 36H, 4C(CH_3_)_3_); 5.29 (s, 2H, 2OH); 6.91 (dd, 2H, Ar, ^3^*J_H-H_* = 8 Hz, ^3^*J_H-H_* = 8 Hz); 7.01 (d, 2H, Ar, ^3^*J_H-H_* = 8 Hz); 7.16 (s, 4H, 2C_6_H_2_); 7.34–7.41 (m, 4H, Ar), 8.60 (s, 2H, 2CH=N). ^13^CNMR (CDCl_3_, 100.6 MHz, *δ*, ppm): 7.52 (Sn-CH_3_); 29.80 (C(CH_3_)_3_); 34.13 (C(CH_3_)_3_); 116.86; 117.48; 118.40; 118.93; 131.55; 132.28; 136.58; 138.71; 146.66; 152.85 (C_Ar_); 159.34 (CH=N). ^119^SnNMR (DMSO-d_6_, 149.15 MHz, *δ*, ppm): −326.37.

#### 3.2.2. Synthesis and Characterization of Bu_2_SnCl_2_(L)_2_ (**2**)

25 mg (0.07 mmol) of Bu_2_SnCl_2_ was added to a solution of 48 mg (0.16 mmol) of **L** in 7 mL of CH_2_Cl_2_. The mixture was stirred at 40 °C for 1.5 h. The solvent was moved away in a vacuum. The resulting oil was treated with hexane. The precipitate formed was rinsed with hexane followed by air drying for 24 h. To obtain crystals suitable for XRD, the substance was recrystallized from hexane. Yield 120 mg (85%);bright orange crystals; m.p. 118–120 °C. Anal. Calcd for C_50_H_72_N_2_O_4_Cl_2_Sn (954.73) (%): C, 62.90; H, 7.60; N, 2.93. Found (%): C, 62.74; H, 7.52; N, 2.65. IR (KBr, ν, cm^−1^): ν(OH) 3492 (s); ν(C-H) 2866–2992 (s); ν(C=N) 1641 (s); 1607; 1487; 1432; 1224; 1105; 756. ^1^H NMR (CDCl_3_, 400.1 MHz, *δ*, ppm): 0.94 (t, 6H, 2CH_2_(CH_2_)_2_CH_3_, ^3^*J_H-H_* = 8 Hz); 1.40–1.46 (m, 4H, 2CH_2_(CH_2_)2CH_3_); 1.49 (s, 36H, 4C(CH_3_)_3_); 1.77–1.84 (m, 8H, 2CH_2_(CH_2_)2CH_3_); 5.30 (s, 2H, 2OH); 6.92 (dd, 2H, Ar, ^3^*J_H-H_* = 8 Hz, ^3^J_H-H_ = 8 Hz); 7.01 (d, 2H, Ar, ^3^*J_H-H_* = 8 Hz); 7.16 (s, 4H, 2C_6_H_2_); 7.34 (dd, 2H, Ar, ^3^*J_H-H_* = 8 Hz, ^3^*J_H-H_* = 8 Hz); 7.39 (d, 2H, Ar, ^3^*J_H-H_* = 8 Hz); 8.60 (s, 2H, 2CH=N). ^13^C NMR (CDCl_3_, 100.6 MHz, *δ*, ppm): 13.09 (CH_3_CH_2_); 25.89 (CH_2_); 26.29 (CH_2_); 26.47 (CH_2_); 29.79 (C(CH_3_)_3_); 34.12 (C(CH_3_)_3_); 116.79; 117.49; 118.42; 119; 131.47; 132.15; 136.55; 139.54; 152.81; 159.38 (C_Ar_); 160.81 (CH=N). ^119^Sn NMR (CDCl_3_, 149.15 MHz, *δ*, ppm): 112.52.

#### 3.2.3. Synthesis and Characterization of Ph_2_SnCl_2_(L) (**3**)

126 mg (0.39 mmol) of **L** was added to a solution of 133 mg (0.39 mmol) of Ph_2_SnCl_2_ in 8 mL of CH_2_Cl_2_. The mixture was stirred at 40 °C for 2 h. The solvent was moved away in a vacuum. The precipitate formed was rinsed with hexane followed by air drying for 24 h.

Yield 258 mg (88%); colouryellow-orange powder;m.p. 76–78°C. Anal. Calcd for C_33_H_37_NO_2_Cl_2_Sn (669.26) (%): C, 59.22; H, 5.57; N, 2.09. Found (%): C, 59.03; H, 5.35; N, 1.85. Selected IR data (KBr, ν, cm^−1^): ν(OH) 3615 (s); ν(C-H) 2873–3052 (s); ν(C=N) 1633 (s); 1608; 1484; 1431; 1226; 1151; 731. ^1^H NMR (CDCl_3_, 400.1 MHz, *δ*, ppm): 1.49 (s, 18H, 2C(CH_3_)_3_); 5.33 (s, 1H, OH); 6.88 (dd, 1H, Ar, ^3^*J_H-H_* = 8 Hz, ^3^*J_H-H_* = 8 Hz); 6.97 (d, 1H, Ar, ^3^*J_H-H_* = 8 Hz); 7.20 (s, 4H, 2C_6_H_2_); 7.33 (dd, 1H, Ar, ^3^*J_H-H_* = 8 Hz, ^3^*J_H-H_* = 8 Hz); 7.38 (d, 1H, Ar, ^3^*J_H-H_* = 8 Hz); 7.52–7.55 (m, 6H, 2Sn-Ph); 7.72–7.75 (m, 4H, 2Sn-Ph, ^2^*J_Sn-H_* = 80 Hz), 8.57 (s, 1H, 2CH=N). ^13^C NMR (CDCl_3_, 100.6 MHz, *δ*, ppm): 29.79 (C(CH_3_)_3_); 34.15 (C(CH_3_)_3_); 117.41; 118.21; 122.51; 128.13; 129.21; 131.25; 131.77; 132.84; 134.71; 136.70; 137.17; 139.81; 153.02 (C_Ar_); 159.06 (CH=N). ^119^Sn NMR (DMSO-d_6_, 149.15 MHz, *δ*, ppm): −267.40.

#### 3.2.4. Synthesis and Characterization of Ph_2_SnCl_2_(L)_2_ (**4**)

107 mg (0.33 mmol) of L and 4 mLof CH_2_Cl_2_ were added to a solution of 57 mg (0.165 mmol) of Ph_2_SnCl_2_ in 4 mLof CH_2_Cl_2_. The mixture was stirred at 40 °C for 3 h. The solvent was moved away in a vacuum. The precipitate formed was rinsed with hexane followed by air-drying for 24 h.

Yield 164 mg (94%); orange powder; m.p.87–89°C. Anal. Calcd for C_54_H_64_N_2_O_4_Cl_2_Sn (994.71) (%): C, 65.20; H, 6.49; N, 2.82. Found (%): C, 64.97; H, 6.34; N, 2.76. IR (KBr, ν, cm^−1^): ν(OH) 3617 (s); ν(C-H) 2872–3053 (s); ν(C=N) 1634 (s); 1612; 1486; 1432; 1228; 1152; 759. ^1^H NMR (CDCl_3_, 400.1 MHz, *δ*, ppm): 1.49 (s, 36H, 4C(CH_3_)_3_); 5.31 (s, 2H, 2OH); 6.90 (dd, 2H, Ar, ^3^*J_H-H_* = 8 Hz, ^3^*J_H-H_* = 8 Hz); 7.00 (d 2H, Ar, ^3^*J_H-H_* = 8 Hz); 7.17 (s, 4H, 2C_6_H_2_); 7.33 (dd, 2H, Ar, ^3^*J_H-H_* = 8 Hz, ^3^*J_H-H_* = 8 Hz); 7.38 (d, 2H, Ar, ^3^*J_H-H_* = 8 Hz); 7.52–7.55 (m, 6H, 2Sn-Ph); 7.72–7.75 (m, 4H, 2Sn-Ph), 8.57 (s, 2H, 2CH=N). ^13^C NMR (CDCl_3_, 100.6 MHz, *δ*, ppm): 29.72 (C(CH_3_)_3_); 34.08 (C(CH_3_)_3_); 116.51; 117.32; 118.39; 118.95; 126.01; 129.29; 131.41; 131.93; 134.82; 136.96; 139.49; 152.80; 159.29 (C_Ar_); 160.49 (CH=N). ^119^Sn NMR (DMSO-d_6_, 149.15 MHz, *δ*, ppm): −237.08.

#### 3.2.5. Synthesis and Characterization of Ph_3_SnBr(L)_2_ (**5**)

64 mg (0.15 mmol) of Ph_3_SnBr was added to a solution of 50 mg (0.155 mmol) of **L** in 4 mL of CHCl_3_. The mixture was stirred at 40 °C for 1 h. Then it was filtered through a paper filter. The solvent from the filtrate was moved away in a vacuum. The precipitate formed was rinsed with hexane followed by air drying for 24 h.

Yield 85 mg (88%); yellow powder; m.p.99–101°C. Anal. Calcd for C_60_H_69_N_2_O_4_BrSn (1080.82) (%): C, 66.68; H, 6.43; N, 2.59. Found (%): C, 66.39; H, 6.31; N, 2.42. IR (KBr, ν, cm^−1^): ν(OH) 3616 (w); ν(C-H) 2872–3067 (s); ν(C=N) 1635 (w); 1613; 1481; 1431; 1234; 1153; 731. ^1^H NMR (CDCl_3_, 400.1 MHz, *δ*, ppm): 1.49 (s, 36H, 4C(CH_3_)_3_); 5.29 (s, 2H, 2OH); 6.92 (dd, 2H, Ar, ^3^*J_H-H_* = 8 Hz, ^3^*J_H-H_* = 8 Hz); 7.01 (d, 2H, Ar, ^3^*J_H-H_* = 8 Hz); 7.16 (s, 4H, 2C_6_H_2_); 7.35 (dd, 2H, Ar, ^3^*J_H-H_* = 8 Hz, ^3^*J_H-H_* = 8 Hz); 7.35–7.41 (m, 4H, 4Ar); 7.46–7.49 (m, 9H, 3Sn-Ph); 7.67–7.70 (m, 6H, 3Sn-Ph), 8.60 (s, 2H, 2CH=N). ^13^C NMR (CDCl_3_, 100.6 MHz, *δ*, ppm): 29.79 (C(CH_3_)_3_); 34.12 (C(CH_3_)_3_); 116.79; 117.50; 118.44; 119.03; 120.68; 128.75; 130.08; 131.45; 132.11; 135.73; 136.41; 136.55; 152.8; 159.41(C_Ar_); 160.73 (CH=N). ^119^Sn NMR (DMSO-d_6_, 149.15 MHz, *δ*, ppm): −262.64.

### 3.3. Crystallographic Data Collection and Structure Determination

All diffraction data were collected on a STOE StadiVari Pilatus 100 K diffractometer (Stoe&Cie, Darmstadt, Germany) [λ(MoKα) = 0.71073 Å, λ(CuKα) = 1.5418 Å, ω-scans] at 293 K [24] The primary processing of the experimental data array was performed using the WinGX program package [25]. The structures were solved by direct methods and refined by full-matrix least-squares procedures on F^2^ using SHELXL97 [26]. All non-hydrogen atoms were refined anisotropically, and hydrogen atoms were located at calculated positions and refined via the ‘riding model’. Crystal data and structure refinement parameters are listed in Table 1. CCDC2205597 (**L**), 2205598 (**2**) contain the supplementary crystallographic data for this paper. The structures of complexes were drawn using the MERCURY CSD 3.1 program [27].

### 3.4. CUPRAC Assay (Cu^2+^ Reducing)

The method proposed by Apak et al. was used with slight modification [14]. For this reason, 0.05 mLof CuCl_2_ solution (0.01 M), 0.05 mLof methanol neocuproine solution (7.5 mM) and 0.25 mLof ammonium acetate buffer solution (1 M) were added to a test tube, followed by mixing with different concentrations of tested compounds (10–100 μM). The mixtures were kept at room temperature. Absorbance was measured at 450 nm on Multiskan Go microplate spectrophotometer (Thermo Fisher Sci., Waltham, MA, USA) against a reagent blank 35 min later. The increase of reaction mixture absorbance in comparison with control indicates the reduction capability of the test compound. Results were presented in Trolox-equivalents. TEAC CUPRAC values were obtained graphically using absorbance data and a linear calibration curve plotted as absorbance vs. Trolox concentration.

### 3.5. DPPH Radical Scavenging Activity

The free radical scavenging activity was evaluated using the stable radical DPPH, according to the method described by Brand-Williams et al. with a slight modification [15]. For each compound, a 1:1 ratio expressed as moles of compound per mole of DPPH radical was tested. A 1 mL sample of the compound solution in ethanol was added to 1 mL of DPPH solution in ethanol so that the initial DPPH concentration in the cuvettes was 0.1 mM. The samples were incubated for 1 h at 20 °C in ethanol and the decrease in the absorbance values of the DPPH solution was measured at λ_max_ 517 nm on the Evolution 300 UV-Visible (Thermo Scientific) cuvette spectrophotometer. Results were expressed as scavenging activity calculated as follows:

Scavenging activity, % = [(A_o_− A_1_)/A_o_] × 100

The concentration of compound needed to decrease 50% of the initial DPPH concentration (EC_50_) is a parameter widely used to measure the antioxidant effect. For determination of the EC_50_ the values of DPPH decrease after 1 h were used. The EC_50_ values were calculated graphically by plotting scavenging activity against compound concentration. Different sample concentrations (0.01, 0.02, 0.04, 0.06, 0.08 and 0.1 mM) were used in order to obtain kinetic curves and to calculate the EC_50_ values.

### 3.6. Lipoxygenase Inhibition Assessment

Lipoxygenase type 1B (LOX-1B) from Glycine max (soybean), boric acid, linoleic acid, ammonium acetate, CuCl_2_, and ethanol (96%) were purchased from Sigma-Aldrich and were used with no further purification. LOX-1B inhibition activity was determined spectrophotometrically [16] by measuring the increase in absorbance at 234 nm for the oxidation of linoleic acid on the Multiskan Go microplate spectrophotometer (Thermo Scientific). The reaction mixture contained 3 µL of test compounds dissolved in DMSO at initial concentrations of 0.05–2 mM (final concentrations 0.8, 3.3, 8.3, 16.6, 33.3 µM); 100 µL of 0.3 mM linoleic acid; 30 µL of borate buffer (pH = 9.0) and 17 µL of lipoxygenase solution in borate buffer. The total sample volume was 150 µL. The total cell volume used for UV-Vis measurements is 300 µL. The increase in absorbance was recorded every 10 s during 10 min under a controlled temperature 25 °C. All experiments were performed in triplicate.

The degree of LOX-1B activity (I %) in the presence of the complexes (Table 5) was calculated according to the following [5]:

I, % = (ν_o_ in the presence of inhibitor/ν_o_ in the absence of inhibitor) × 100,

where ν_o_ is the initial rate. The value of the initial rate (ν_o_, µM min^−1^) was calculated according to the formula:

ν_o_ = ∆C/∆t = ∆A/∆tε = tgα/∆tε,

where C is the concentration of product (hydroperoxy-linoleic acid), t is the reaction time, ε is the molar absorbance coefficient of hydroperoxy-linoleic acid and tgα is the slope of the kinetic curve plotted as absorbance vs. time.

### 3.7. Antiproliferative Activity [28]

The human HCT116 colorectal carcinoma, A549 non-small cell lung carcinoma, MCF7 breast adenocarcinoma cell lines and WI-38 diploid human cell line composed of fibroblasts were obtained from the European collection of authenticated cell cultures (ECACC; Salisbury, UK) All cells were grown in a DMEM medium (Gibco™, Ireland) supplemented with 10% fetal bovine serum (Gibco™, Brazil). The cells were cultured in an incubator at 37 °C in a humidified 5% CO_2_ atmosphere and were sub-cultured 2 times a week. The effect of the investigated compounds on cell proliferation was evaluated using a common MTT assay. The cells were seeded in 96-well tissue culture plates («TPP», Trasadingen, Switzerland) at 7 × 10^3^ cells/well in 100 µL of the medium. After overnight incubation at 37 °C, the cells were treated with the tested compounds in the concentration range of 0 to 100 µM. Cisplatin was used as a standard. After 72 h of treatment, the solution was removed, and a freshly diluted MTT solution (100 µL, 0.5 mg/mL in cell medium) was added to the wells, and the plates were further incubated for 50 min. Subsequently, the medium was removed, and the formazan product was dissolved in 100 μL of DMSO. The number of living cells in each well was evaluated by measuring the absorbance at 570 nm using the «Zenith 200 rt» microplate reader (Biochrom, Cambridge, UK) The meanings of 50% inhibition concentration (IC_50_) with standard deviation were calculated using GraphPad Prism Version 5.03 for Windows.

### 3.8. Cell Death and Cell Cycle Analysis

HCT-116 cells (colon carcinoma) (1 × 10^6^) were seeded in a 6-well plate and incubated with **2**, **5** and cisplatin (values based on MTT assay) at 2 × IC_50_ for 24 and 48 h. After incubation, the cells were harvested by trypsinization, precipitated by centrifugation (3500 rpm), washed with cold PBS and recentrifuged. Aliquots of cells were processed as recommended in the Muse AnnexinV&Dead Cell Kit. The results were recorded on a Muse Cell Analyzer flow cytometer (Luminex corp., Austin, TX, USA) [20].

For the cell cycle analysis, HCT-116 cells (1 × 10^6^) were seeded in a six-well plate and were incubated for 24 h and then the medium was treated with compounds at the concentrations IC_50_ and incubated for 24 h. After incubation, the cells were harvested by trypsinization and precipitated by centrifugation (3500 rpm). After precipitation, the supernatant was removed, washed with PBS, centrifuged, fixed with 70% ethanol and incubated for at least 3 h at −20 °C. After incubation, 200 µL of the cell suspension was collected, centrifuged, the supernatant was removed, and washed with 200 µL of PBS. Then, the cells were stained 200 µL of the Muse Cell Cycle Reagent and incubated for 30 min at r.t. in the dark. Cell cycle analysis was performed using a Muse Cell Analyzer flow cytometer (Merck, Rahway, NJ, USA).

### 3.9. Tubulin Polymerization

Evaluation of the effect of compounds on tubulin polymerization performed according to [29]. A crude fraction of tubulin and microtubule-associated proteins (Tb+MAP) was isolated from mouse brain tissue by a polymerization–depolymerization method [30].

The procedure of mouse euthanasia using cervical dislocation for Tb+MAP preparation is in compliance with the Guidelines for Animal Experiments, which were approved by the local bioethics committee of the IPAC RAS. The freshly dissected brain was immediately placed on ice, cleared of meninges and surface blood vessels, and washed with cold buffer A (50 mM Tris-HCl, pH 6.9 at 4 °C, 2 mM EGTA). The extracted brain tissue was homogenized under ice cooling in the same buffer using a Potter S homogenizer (Sartorius, Göttingen, Germany). The resulting homogenate was centrifuged at 10,000× *g* for 30 min on an Avanti 25 centrifuge (Beckman, Brea, CA, USA) using a JA-14 rotor for the precipitation of non-ruptured cells. The precipitate was discarded, and the supernatant was again centrifuged at 100,000× *g* for 60 min at 4 °C on an Optima MAX XP centrifuge (Beckman, Brea, CA, USA) using a MLA-50 rotor. The precipitate was again discarded. The protein concentration in the supernatant was determined by the Bradford assay using the Bio-Rad Protein Assay kit (Bio-Rad, Hercules, CA, USA) according to the manufacturer’s instructions. The resulting supernatant was a cytosolic fraction enriched in microtubular proteins (Tb and MAP). The Tb+MAP preparation was centrifuged at 5000× *g* for 10 min at 4 °C immediately before polymerization in order to remove denatured and aggregated protein molecules by precipitation. The Tb+MAP polymerization was performed at 37 °C in buffer A in the presence of 0.1 mM GTP after the addition of 100 M of test compound or the same volume of vehicle (DMSO). The protein concentration in the sample was 0.2 mg/mL. The polymerization kinetics was monitored on a Victor 3 or EnVision microplate reader (Perkin Elmer, Waltham, MA, USA) at 355 nm.

## 4. Conclusions

Novel polyfunctional organotin complexes **1**–**5** based on a Schiff base containing an antioxidant 2,6-di-*tert*-butylphenol moiety were obtained and characterized. Using XRD method, the structures of initial ligand **L** and compound **2** were defined. In complex **2** the ligand is coordinated by O atom to the Sn center and distorted octahedron geometry was observed. In addition, a proton transfer from the oxygen atom to the nitrogen atom in the ligand part was revealed for complex **2**.

Complexes **1** and **5** demonstrated high antioxidant activity in CUPRAC- and DPPH-methods due to their ability to be involved in one-electron and hydrogen atom transfer. The antioxidant activity was also explored in the enzymatic oxidation of linoleic acid by lipoxygenase (LOX 1-B) in vitro. Only compound **4** was found to be a moderate inhibitor of LOX 1-B.

The antiproliferative activity of complexes **1**–**5** was studied in vitro on HCT-116, MCF-7, A-549 and WI-38 cancer cell lines in the MTT test. It was established that dibutyl- and triphenyltin complexes **2** and **5** were the most active. Apoptosis and the cell cycle analysis were investigated by flow cytometry on HCT-116 cell line for these compounds after 24 and 48 h. Complex **2** was found to be the most active compound inducing apoptosis. Moreover, compounds **2** and **5** blocked proliferation in the G2/M phase of the cell cycle. The mechanism of antiproliferative activity of compounds **2** and **5** may be also connected with their influence on Tb+MAP polymerization.

Thus, the obtained compounds demonstrate high biological activity, which makes thempromising for further research as potential anti-tumor agents with possible combined actions.

## Figures and Tables

**Table 1:** Crystal data and the structure refinement details for compounds **L** and **2**.

Compound	L	2
Empirical formula	C_21_H_27_NO_2_	C_25_H_36_ClNO_2_Sn_0_._50_
Fw T, K	325.43 293(2)	477.33 293(2)
Space group	*P*2_1_2_1_2_1_	*P*2_1_/*n*
Syngony	Orthorhombic	Monoclinic
*a* (Å)	6.0356(4)	9.5216(5)
*b* (Å)	17.5484(14)	11.7902(7)
*c* (Å)	17.7780(12)	22.4464(11)
*α* (°)	90.00	90.00
*β* (°)	90.00	98.820(3)
*γ* (°)	90.00	90.00
*V* (Å^3^)	1883.0(2)	2490.1(2)
*Z* λ	4 MoKα	4 CuKα
Δρ_max_/Δρ_min_(*e*/Å^3^)	0.212/−0.210	2.028/−1.732
*μ* (mm^−1^)	0.073	5.396
GOOF	0.864	1.122
*R*_1_/*wR*_2_(*I* ≥ 2σ(*I*)	0.0458/0.1172	0.0772/0.2112

**Table 2:** Selected bond distances (Å) and angles (°) for compounds **L** and **2**. Symmetry operation i = −x, 1 − y, 1 − z.

L		2	
Selected bond distances (Å)			
O(1)-C(4) O(2)-C(13) N(1)-C(7) N(1)-C(1)	1.365(3) 1.342(3) 1.272(3) 1.419(3)	Sn(1)-C(22) Sn(1)-O(2) Sn(1)-Cl(1)	2.140(7) 2.258(4) 2.6123(18)
Angles (°)			
C(4)-O(1)-H(31) C(13)-O(2)-H(32) C(7)-N(1)-C(1) C(7)-N(1)-H(32) C(1)-N(1)-H(32) C(6)-C(1)-N(1) C(2)-C(1)-N(1) O(1)-C(4)- C(5) O(1)-C(4)-C(3) N(1)-C(7)-C(8) N(1) C(7)-H(7) O(2)-C(13)-C(12) O(2)-C(13)-C(8)	113(2) 103.9(17) 123.4(2) 101.4(14) 134.8(14) 116.1(2) 124.5(2) 115.2(2) 122.6(2) 121.8(2) 124.3(14) 119.6(2) 120.5(2)	C(22)-Sn(1)-O(2) C(22)^i^-Sn(1)-O(2) C(22)-Sn(1)-O(2)^i^ C(22)^i^-Sn(1)-O(2)^i^ O(2)-Sn(1)-O(2)^i^ C(22)-Sn(1)-Cl(1) C(22)^i^ -Sn(1)-Cl(1) O(2)-Sn(1)-Cl(1) O(2)^i^ -Sn(1)-Cl(1) C(22)-Sn(1)-Cl(1)^i^ C(22)^i^ -Sn(1)-Cl(1)^i^ O(2)-Sn(1)-Cl(1)^i^ O(2)^i^ -Sn(1)-Cl(1)^i^	88.31(19) 91.69(19) 91.69(19) 88.31(19) 180.00(18) 91.89(15) 88.11(15) 92.11(12) 87.89(12) 88.11(15) 91.89(15) 87.89(12) 92.11(12)

**Table 3:** The TEAC values of Cu^2+^ reducing activity in CUPRAC test for compounds **L**, **1**–**5**.

Compound	TEAC (CUPRAC)
L	0.81 ± 0.03 [13]
1	3.37 ± 0.03
2	2.78 ± 0.04
3	1.19 ± 0.09
4	2.64 ± 0.07
5	2.31 ± 0.06

Results presented as mean ± SD obtained using data from at least three independent experiments.

**Table 4:** EC_50_ and *k* values in the DPPH test (EtOH, 20°C) in the presence of **1**–**5**.

Compound		1	2	3	4	5
EC_50_, µM		16 ± 2	20 ± 3	30 ± 3	19 ± 2	23 ± 2
s		0.32	0.4	0.30	0.38	0.23
s^−1^		3.13	2.5	3.36	2.60	4.32
C	*k* (L mol^−1^ s^−1^)					
	0.06 mM	*	49 ± 5	*	*	*
	0.04 mM	31 ± 4	29 ± 3	5 ± 1	49 ± 4	10 ± 1
	0.02 mM	13 ± 4	2 ± 0.5	1 ± 0.2	3 ± 0.5	3 ± 0.4
	0.01 mM	1 ± 0.3	0.7 ± 0.1	0.9 ± 0.1	2 ± 0.1	0.7 ± 0.1

s = 2∙EC_50_/C_0(DPPH)_—the number of moles of compound required to reduce 1 mole of DPPH; s^−1^—the number of moles of DPPH reduced by 1 mole of the compound; the initial concentration of DPPH is 0.1 mM. Results presented as mean ± SD obtained using at least from three independent experiments.*—the parameter is not defined due to the high speed of the reaction.

**Table 5:** EC_50_ (μM) and degree of inhibition (I, %) of LOX 1-B lipoxygenase in the presence of compound **2**.

C, μM	I, %
0.8	22.66
8.3	27.59
16.6	31.21
33.3	39.55

**Table 6:** IC_50_ values (µM) against HCT-116, MCF-7, A-549 and WI-38 cell lines for compounds **1**–**5** and cisplatin.

Compound		IC_50_, µM		
	HCT-116	MCF-7	A-549	WI-38
1	58.2 ± 14	80.6 ± 6	89 ± 17	58.2 ± 8
2	1.6 ± 0.2	4.3 ± 0.5	5.7 ± 1	1.6 ± 0.2
3	3.8 ± 0.5	6.3 ± 0.7	7.7 ± 1.4	2.3 ± 0.3
4	3.1 ± 0.4	5.1 ± 0.6	6.8 ± 1.8	2.1 ± 0.3
5	0.32 ± 0.08	0.29 ± 0.03	0.30 ± 0.04	0.2 ± 0.05
cisplatin	9.04 ± 0.7	11 ± 1	16.7 ± 3	4.8 ± 0.5

Results presented as mean ± SD obtained using at least from three independent experiments.

## Data Availability

Copies of ^1^H, ^13^C, ^119^Sn NMR, ECI-MS spectra and structure files of the synthesized compounds are presented in the Supplementary Materials.

## Supplementary Materials

The following supporting information can be downloaded at: https://www.mdpi.com/article/10.3390/ijms24032024/s1.
