# Supplementary material for: Biological Activity of Novel Organotin Compounds with a Schiff Base Containing an Antioxidant Fragment
Source: Int J Mol Sci. 2023 Jan 19;24(3):2024. doi: 10.3390/ijms24032024 (PMC9916890; doi:10.3390/ijms24032024)
Supplement: Supplementary file 1 [file ijms-24-02024-s001.zip › ijms-2158172-supplementary.pdf]

Supplementary Materials

for

Biological Activity of Novel Organotin Compounds with a Schiff Base Containing an Antioxidant Fragment

Taisiya A. Antonenko[1], Yulia A. Gracheva[1], Dmitry B. Shpakovsky[1], Mstislav A. Vorobyev[2], Dmitrii M. Mazur[1], Victor A. Tafeenko[1], Yury F. Oprunenko[1], Elena F. Shevtsova[3], Pavel N. Shevtsov[3], Alexey A. Nazarov[1,*]
and Elena R. Milaeva[1]

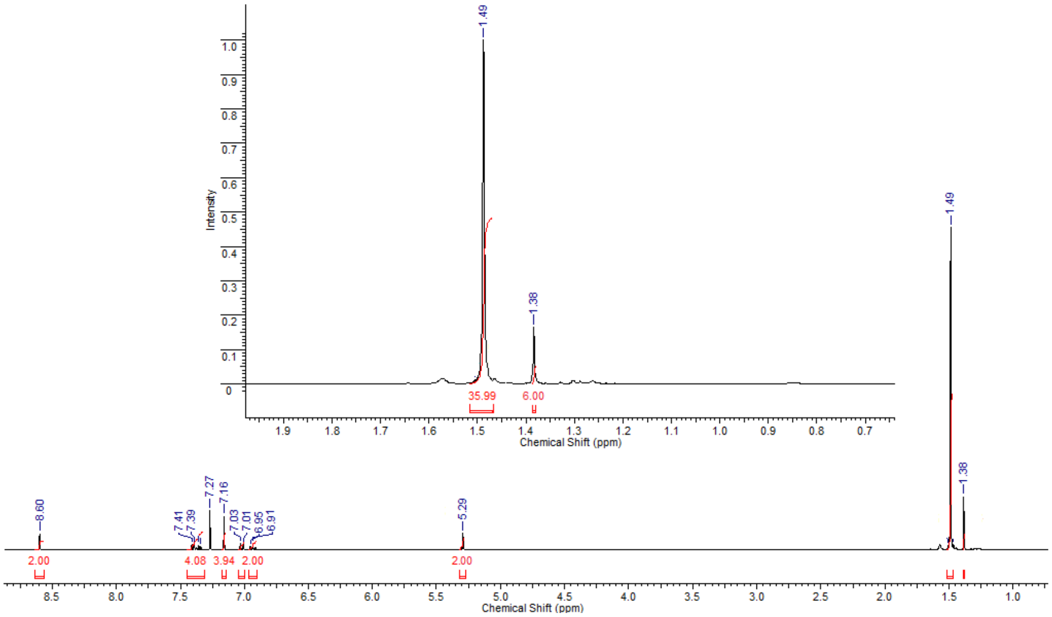

Figure S1. The ^{1}H NMR spectra of **1**.

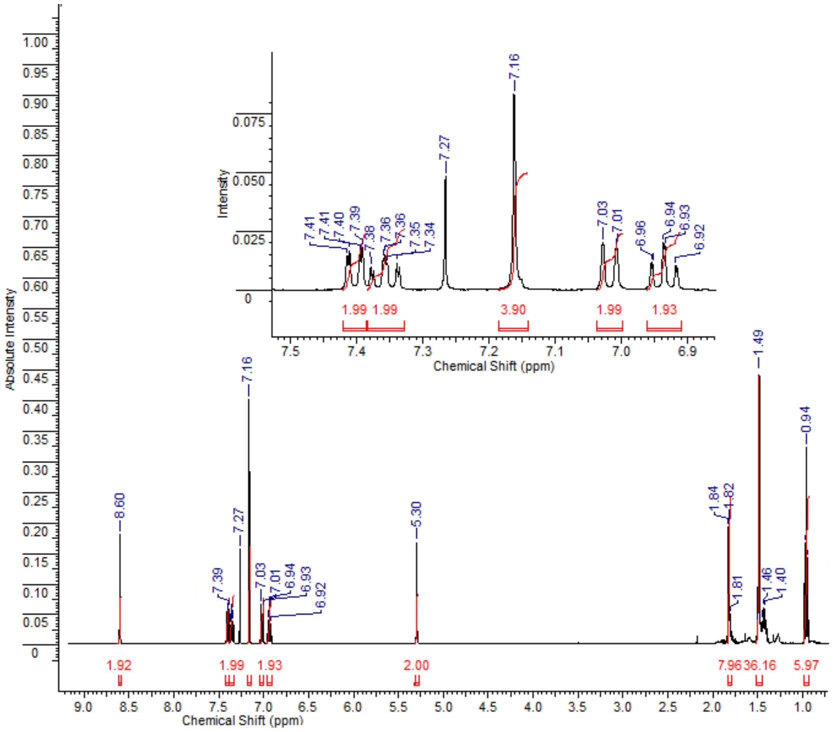

Figure S2. The ^{1}H NMR spectra of **2**.

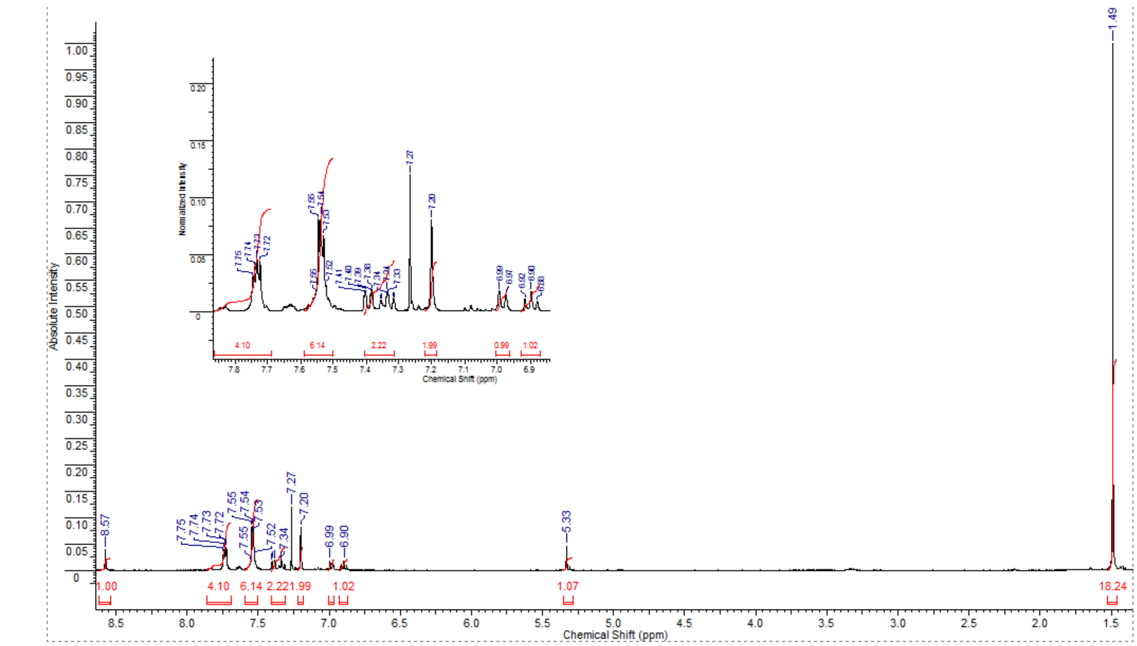

Figure S3. The ^{1}H NMR spectra of **3**.

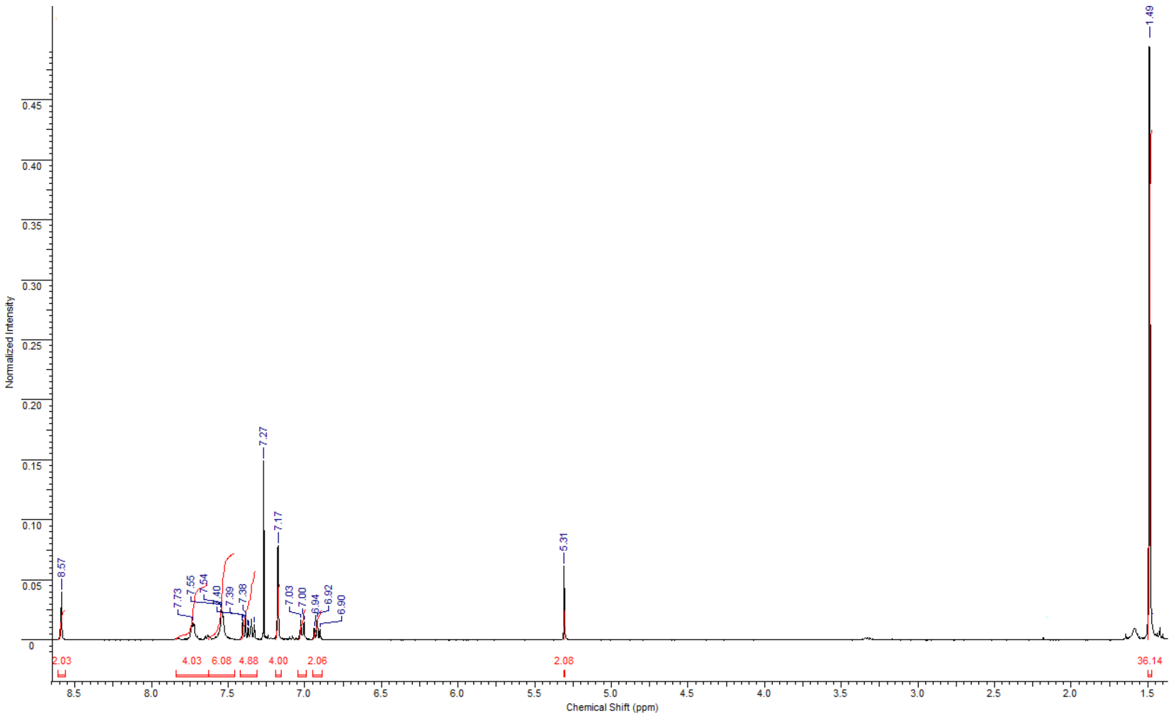

Figure S4. The ^{1}H NMR spectra of **4**.

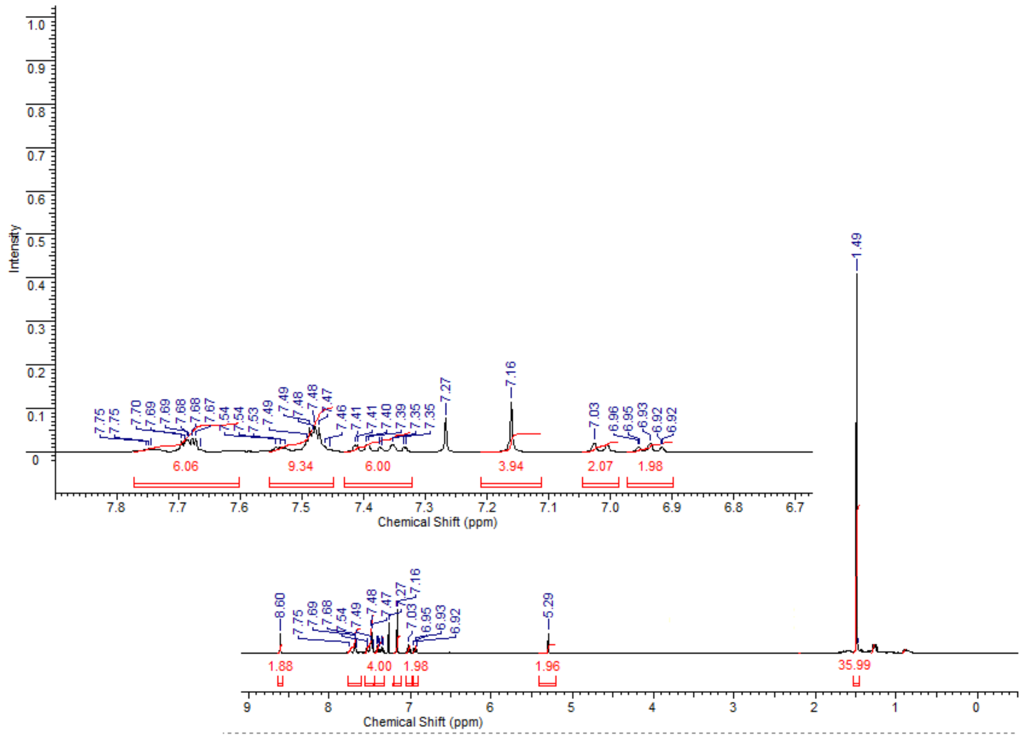

Figure S5. The ^{1}H NMR spectra of **5**.

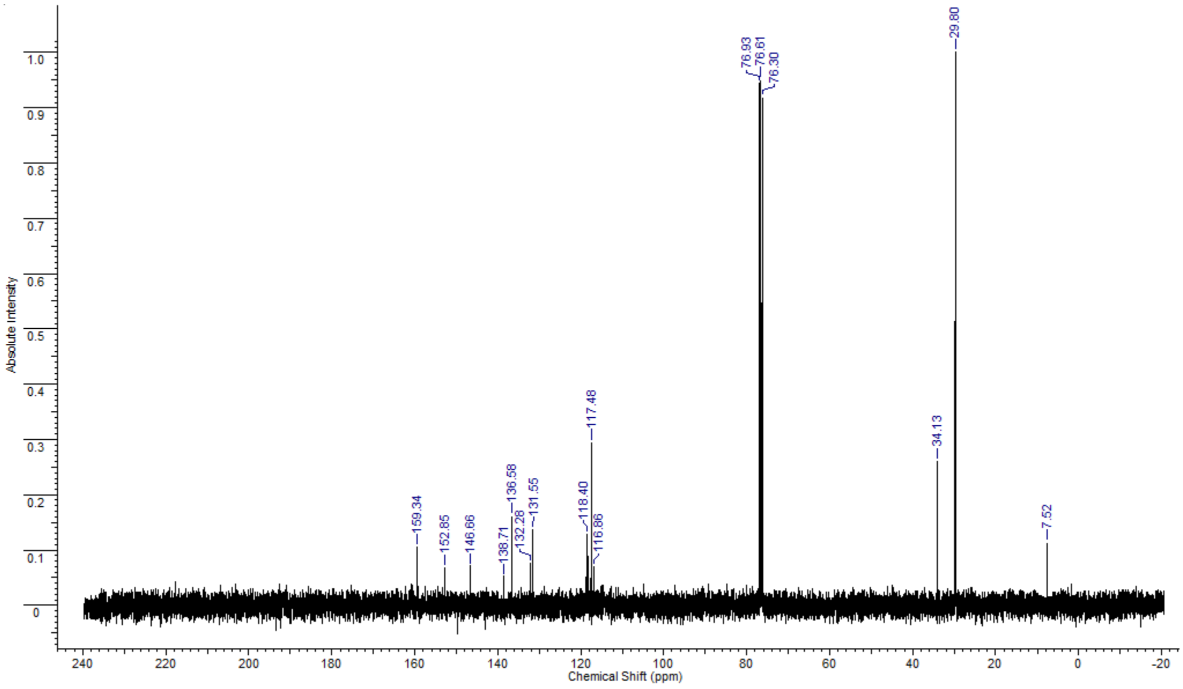

Figure S6. The ^{13}C NMR spectra of **1**.

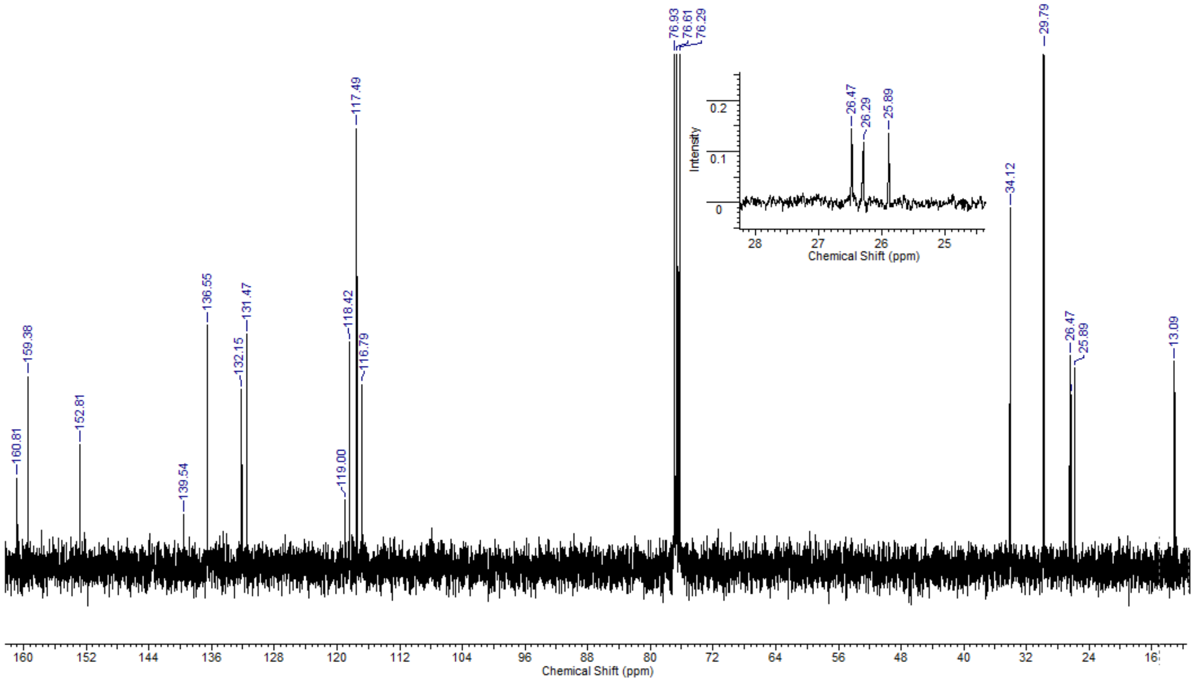

Figure S7. The ^{13}C NMR spectra of **2**.

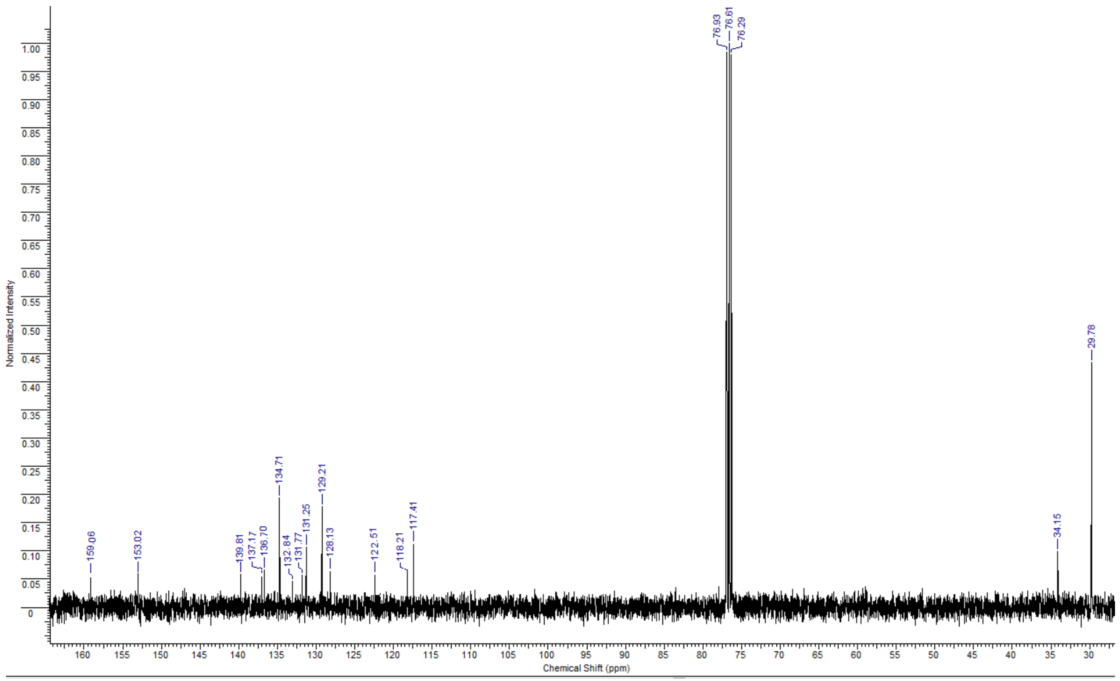

Figure S8. The ^{13}C NMR spectra of **3**.

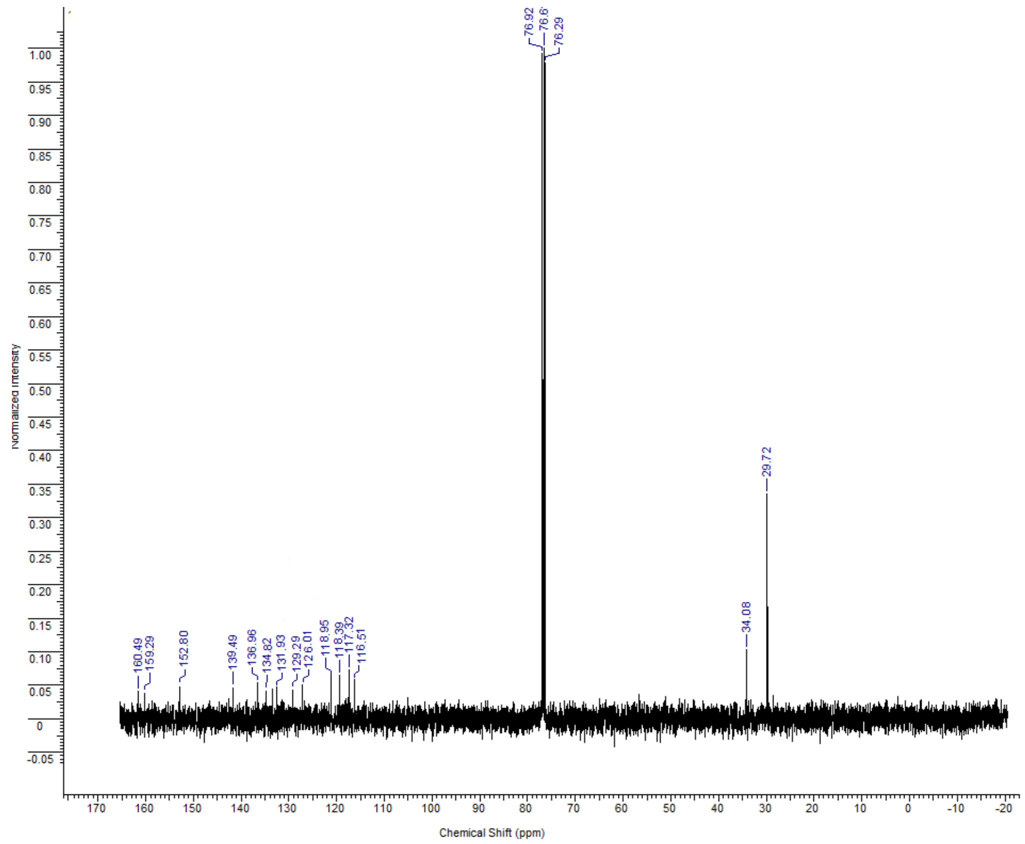

Figure S9. The ^{13}C NMR spectra of **4**.

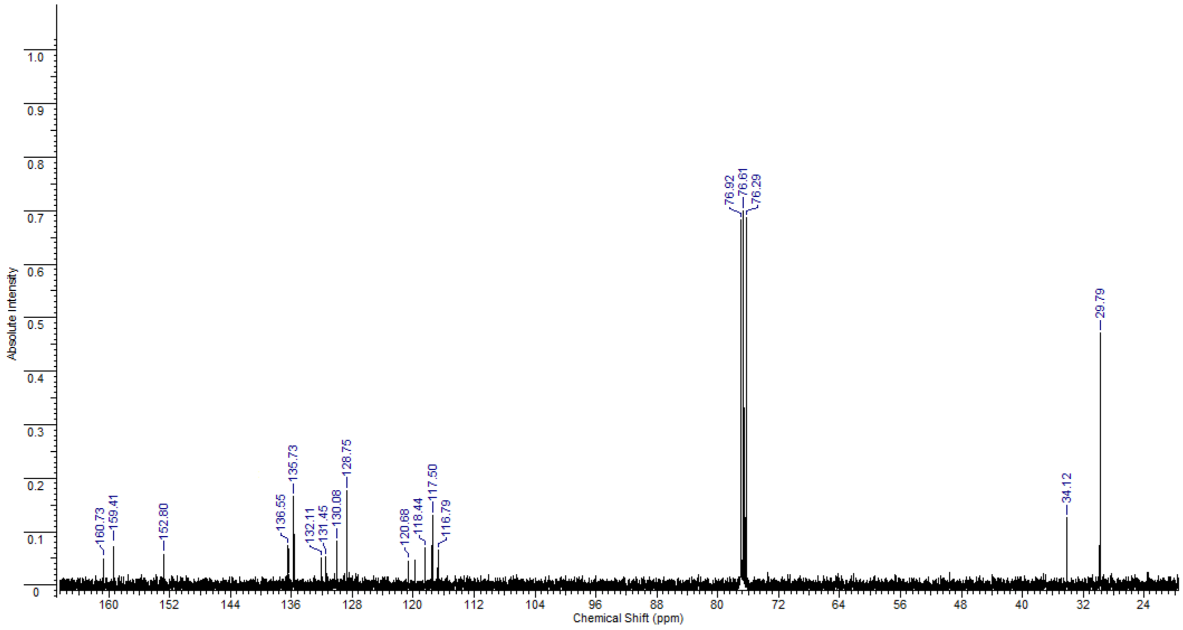

Figure S10. The ^{13}C NMR spectra of **5**.

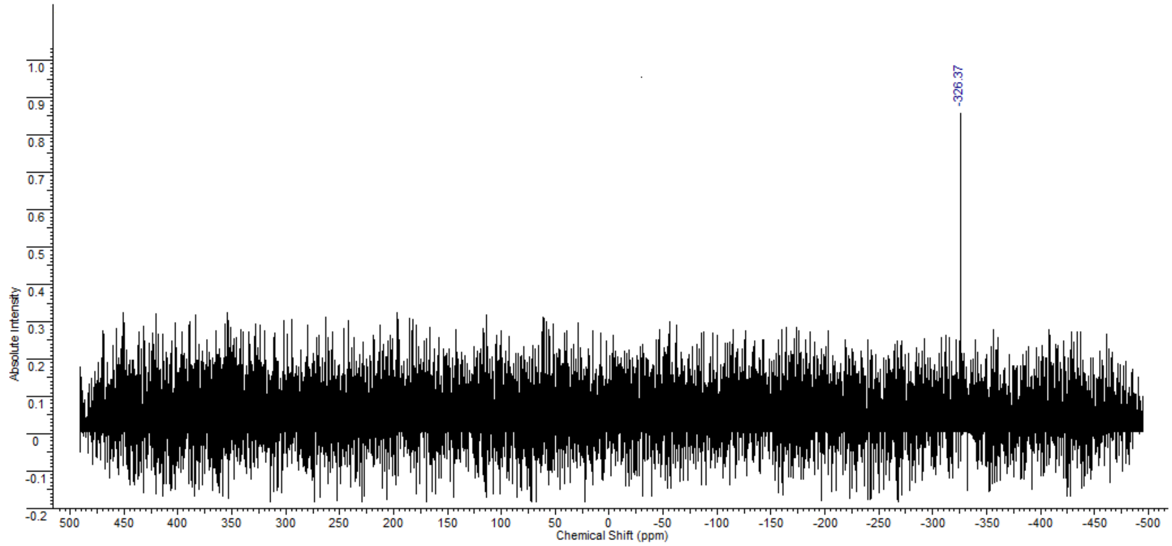

Figure S11. The ^{119}Sn NMR spectra of **1**.

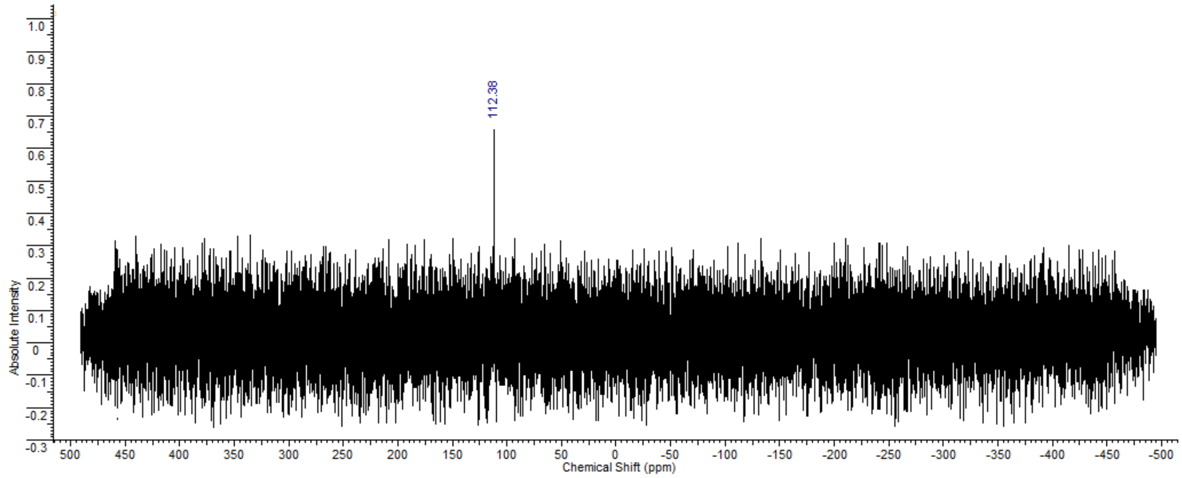

Figure S12. The ^{119}Sn NMR spectra of **2**.

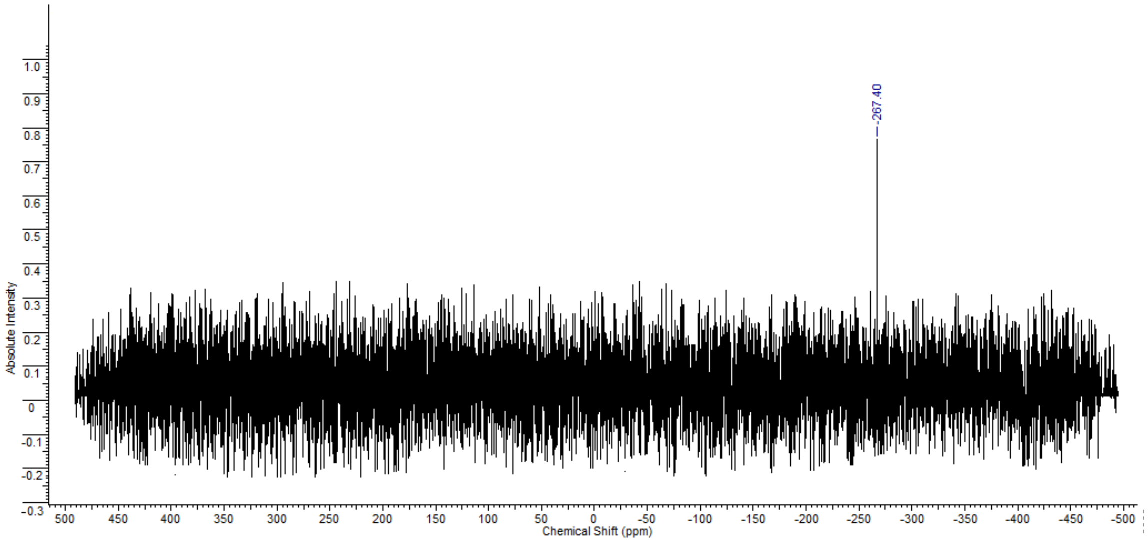

Figure S13. The ^{119}Sn NMR spectra of **3**.

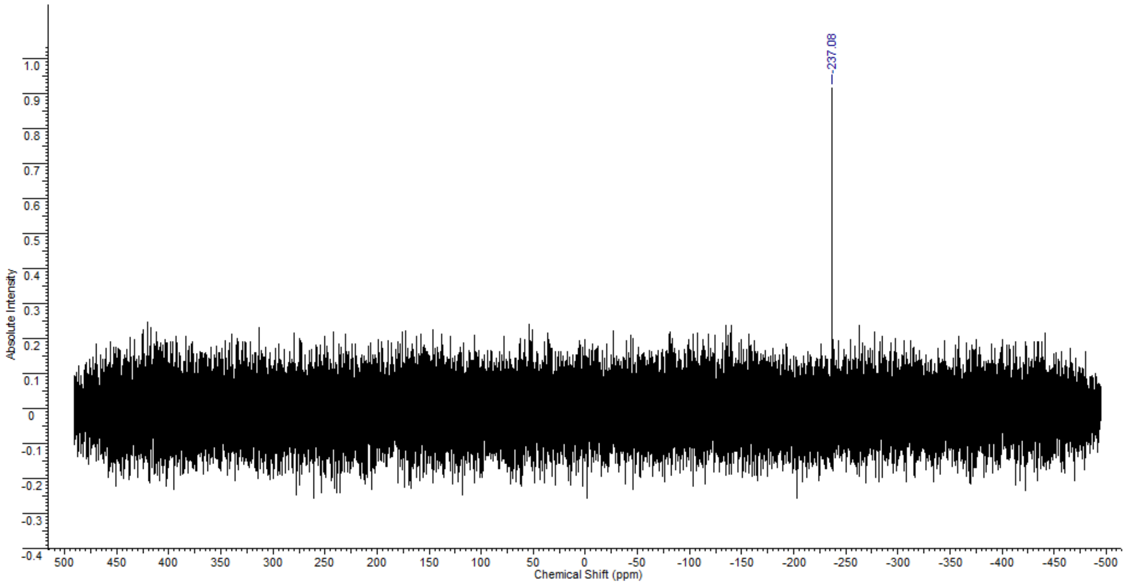

Figure S14. The ^{119}Sn NMR spectra of **4**.

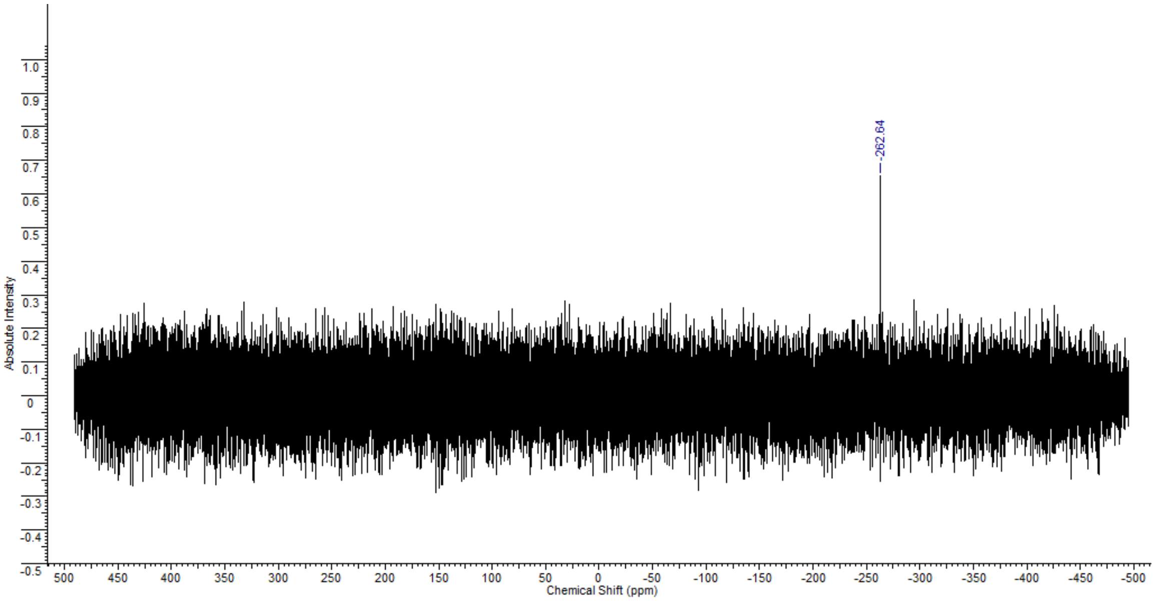

Figure S15. The ^{119}Sn NMR spectra of **5**.

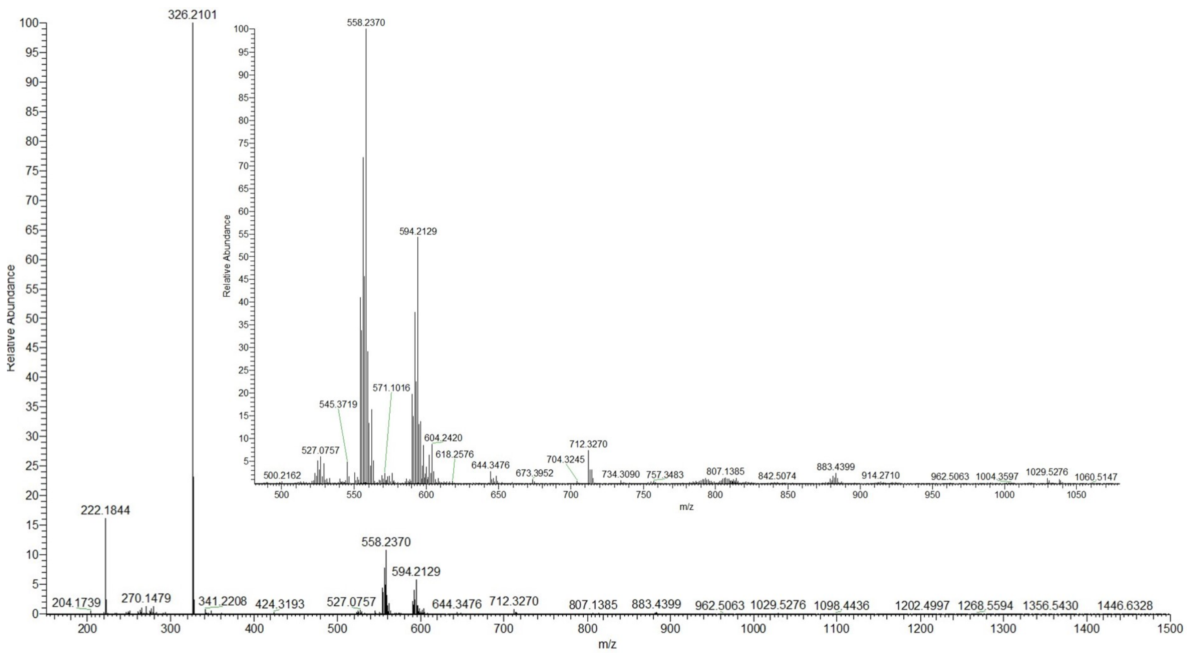

Figure S16. ESI mass spectrum of **2**.

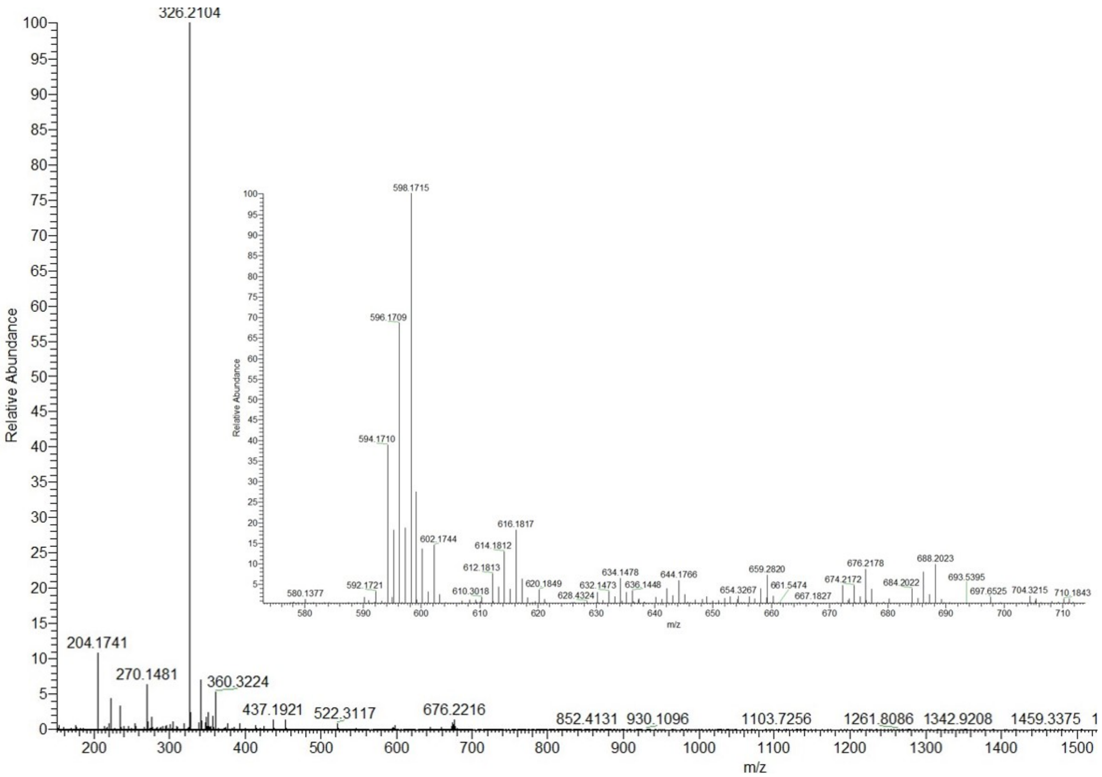

Figure S17. ESI mass spectrum of **3**.

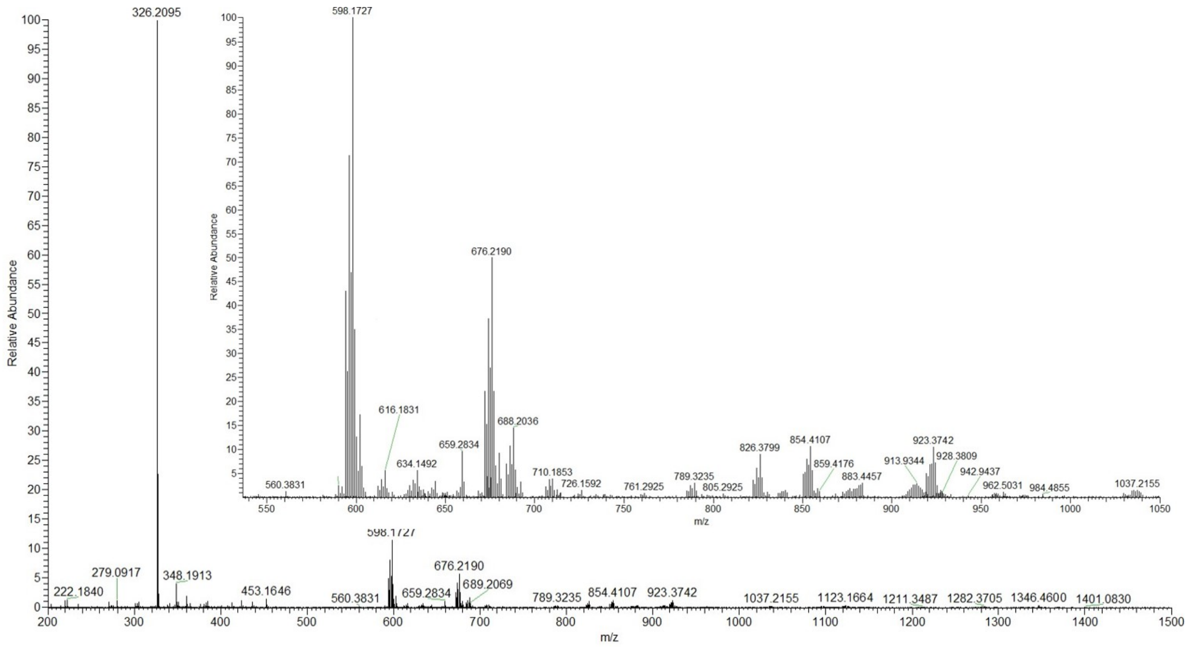

Figure S18. ESI mass spectrum of **4**.

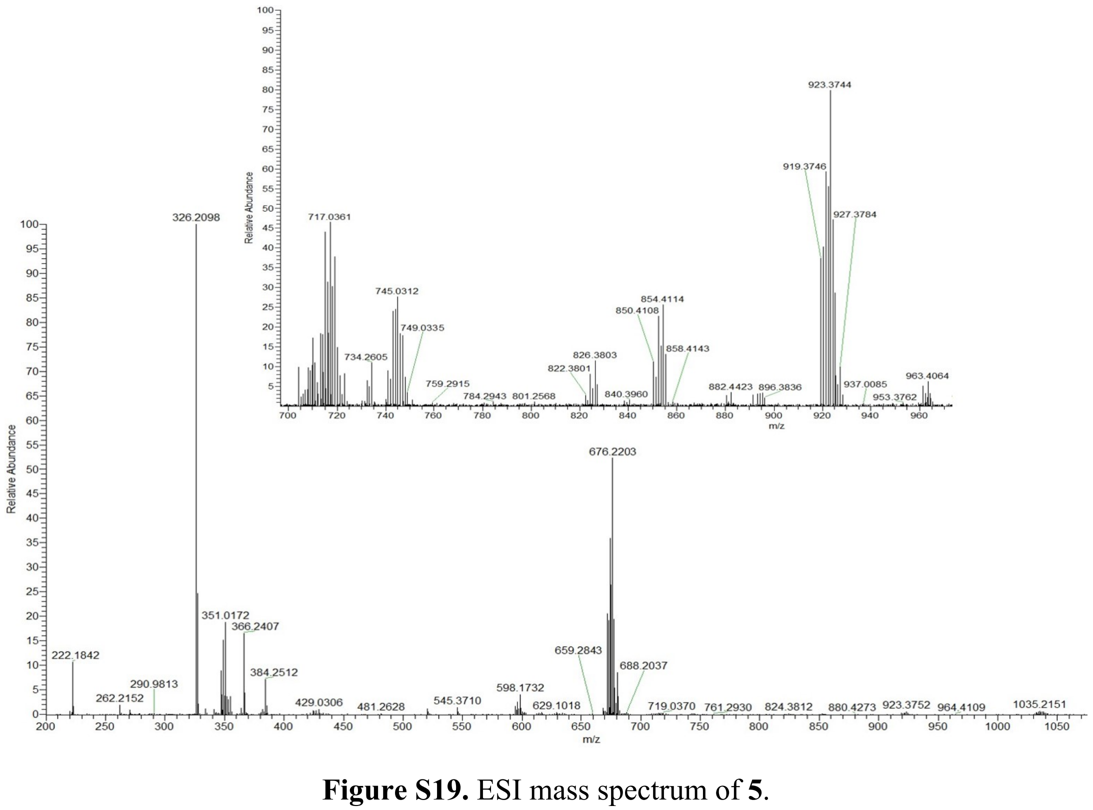

Figure S19. ESI mass spectrum of **5**.
